# Effective connectivity: Influence, causality and biophysical modeling

**DOI:** 10.1016/j.neuroimage.2011.03.058

**Published:** 2011-09-15

**Authors:** Pedro A. Valdes-Sosa, Alard Roebroeck, Jean Daunizeau, Karl Friston

**Affiliations:** a Cuban Neuroscience Center, Ave 25 #15202 esquina 158, Cubanacan, Playa, Cuba; b Department of Cognitive Neuroscience, Faculty of Psychology and Neuroscience, Maastricht University, The Netherlands; c The Wellcome Trust Centre for Neuroimaging, Institute of Neurology, UCL, 12 Queen Square, London, WC1N 3BG, UK; d Laboratory for Social and Neural Systems Research, Institute of Empirical Research in Economics, University of Zurich, Zurich, Switzerland

**Keywords:** Granger Causality, Effective connectivity, Dynamic Causal Modeling, EEG, fMRI

## Abstract

This is the final paper in a Comments and Controversies series dedicated to “The identification of interacting networks in the brain using fMRI: Model selection, causality and deconvolution”. We argue that discovering effective connectivity depends critically on state-space models with biophysically informed observation and state equations. These models have to be endowed with priors on unknown parameters and afford checks for model Identifiability. We consider the similarities and differences among Dynamic Causal Modeling, Granger Causal Modeling and other approaches. We establish links between past and current statistical causal modeling, in terms of Bayesian dependency graphs and Wiener–Akaike–Granger–Schweder influence measures. We show that some of the challenges faced in this field have promising solutions and speculate on future developments.

## Introduction

Following an empirical evaluation of effective connectivity measurements (David et al., 2008) and a primer on its implications (Friston, 2009a), the Comments and Controversy (C&C) exchange, initiated by Roebroeck et al. (2011b-this issue) and continued by David, 2009, Friston, 2011, and Roebroeck et al. (2011a-this issue), has provided a lively and constructive discussion on the relative merits of two current techniques, Granger Causal Modeling (GCM)^1^ and Dynamic Causal Modeling (DCM), for detecting effective connectivity using EEG/MEG and fMRI time series. The core papers of the C&C have been complemented by authoritative contributions (Bressler and Seth, 2010, Daunizeau et al., 2011a, Marinazzo et al., 2010) that clarify the state of the art for each approach.

**Fig. 1:**
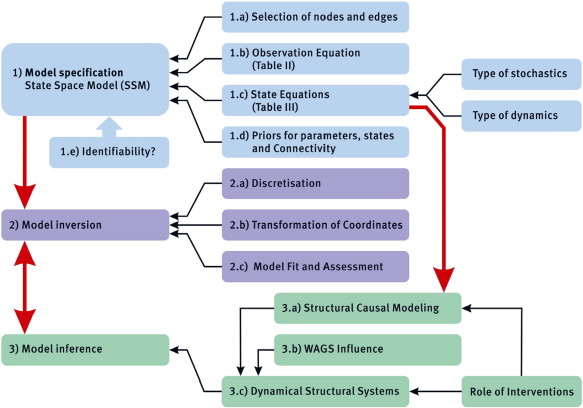
Overview of causal modeling in Neuroimaging. Overall view of conceptual framework for defining and detecting effective connectivity in Neuroimaging studies.

**Fig. 2:**
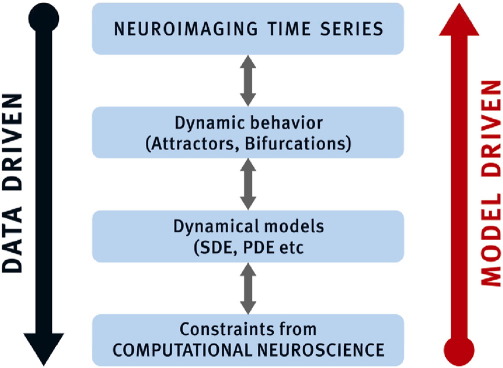
Data and model driven approaches to causal modeling. Data driven approaches look for nonparametric models that not only fit the data but also describe important dynamical properties. They complement hypothesis driven approaches that are not only constrained by having to explain dynamical behavior but also provide links to computational models of brain function.

This final paper in the series attempts to summarize the main points discussed and elaborate a conceptual framework for the analysis of effective connectivity (Fig. 1, Fig. 2). Inferring effective connectivity comprises the successive steps of model specification, model identification and model (causal) inference (see Fig. 1).These steps are common to DCM, GCM and indeed any evidence-based inference. We will look at the choices made at each stage to clarify current areas of agreement and disagreement, of successes and shortcomings.

This entails a selective review of key issues and lines of work. Although an important area, we will not consider models that are just used to measure statistical associations (i.e. functional connectivity). In other words, we limit our focus to discovering effective connectivity (Friston, 2009a); that is causal relations between neural systems. Importantly, we hope to establish a clear terminology to eschew purely semantic discussions, and perhaps dispel some confusion in this regard. While preparing this material, we were struck with how easy it is to recapitulate heated arguments in other fields (such as econometrics), which were resolved several decades ago. We are also mindful of the importance of referring to prior work, to avoid repeating past mistakes^2^ and to identify where more work is needed to address specific problems in the neurosciences.

We shall emphasize several times in this paper that causality is an epistemological concept that can be particularly difficult to capture with equations. This is because one's intuitive understanding of causality becomes inherently constrained whenever one tries to model it. In brief, one can think of causality in at least two distinct ways:

• Temporal precedence, i.e.: causes precede their consequences;
• Physical influence (control), i.e.: changing causes changes their consequences.

This distinction is important, since it is the basis for any statistical detection of causal influence. In the context of brain connectivity, identifying causal relationships between two regions in the brain thus depends upon whether one tests for improvement in predictive capacity between temporally distinct neural events or one assesses the distal effect of (experimentally controlled) interventions.

Temporal precedence is the basis for Granger-like (what we call WAGS influence, see WAGS influence section) inferences about causality. In its simplest form, the idea is the following: A WAGS-causes B if one reduces the uncertainty about the future of B given the past of A. Statistical tests of WAGS-causality thus rely upon information theoretic measures of predictability (of B given A).

In contradistinction, physical influence speaks to the notion of intervention and control, which has been formalized using a probabilistic framework called causal calculus (Pearl, 2000) (Structural causal modeling: graphical models and Bayes–Nets section). Observing (or estimating) activity at a network node potentially provides information about its effects at remote nodes. However, physically acting upon (e.g., fixing) this activity effectively removes any other physical influence this node receives. This means that inferences based on the effects of an intervention are somewhat different in nature from those based on purely observational effects. Generally speaking, inference on structural causality rests on modeling the effects of (controlled) experimental manipulations of the system, c.f. the popular quote ‘no causes in, no causes out’ (Cartwright, 2007). As we shall see later, these two approaches can be combined (Dynamic structural causal modeling section).

The structure of the paper is as follows. We first review the types of models used for studying effective connectivity. We then touch briefly on the methods used to invert and make inferences about these models. We then provide a brief summary of modern statistical causal modeling, list some current approaches in the literature and discuss their relevance to brain imaging. Finally, we list outstanding issues that could be addressed and state our conclusions.

## Model specification

### State-space models of effective connectivity

From the C&C discussion, there seems to be a consensus that discovering effective connectivity in Neuroimaging is essentially a comparison of generative models based on state-space models (SSM) of controllable (i.e., “causal” in a control theory sense) biophysical processes that have hidden neural states and possibly exogenous input. While having a long history in engineering (Candy, 2006, Kailath, 1980), SSM was only introduced recently for inference on hidden neural states (Valdes-Sosa et al., 1999, Valdes-Sosa et al., 1996, Valdés-Sosa et al., 1996). For a comprehensive review of SSM and its application in Neuroscience see the forthcoming book (Ozaki, 2011).

Neural states describe the activity of a set of “nodes” that comprise a graph, the purpose of causal discovery being the identification of active links (edges or connections) in the graph. The nodes can be associated with neural populations at different levels; most commonly at the macroscopic (whole brain areas) or mesoscopic (sub-areas to cortical columns) level. These state-space models have unknown parameters (e.g., effective connectivity) and hyperparameters (e.g., the amplitude of random fluctuations). The specific model, states, parameters, hyperparameters and observables chosen determines the type of analysis and the nature of the final inference about causality. These choices are summarized in Fig. 1 (Step 1).

Given a set of observations or brain measurements, the first problem is: which data features are relevant for detecting causal influences? The most efficient way to address this question is to specify a *generative model*, i.e. a set of equations that quantify how observed data are affected by the presence of causal links. Put simply, this model translates the assumption of (i) *temporal precedence* or (ii) *physical influence* into how data should appear, given that (i) or (ii) is true. By presenting the data to generative models, model comparison can then be used to decide whether some causal link is likely to be present (by comparing models with and without that link). We now turn to the specification of generative models, in the form of a SSM.

### Nodes and random variables

The first things we consider are the basic units or nodes, among which one wants to find causal links. These are usually modeled as macroscopic, coarse grained, ensembles of neurons, whose activity is summarized by a time varying state vector *x*_*r*_(*t*) or *x*(*r*, *t*): *r* ∈ *R*. For example *x*(*t*) could be instantaneous (ensemble average) post-synaptic membrane depolarization or pre-synaptic firing rate of neurons. The set *R* of nodes is usually taken as a small number of neural masses corresponding to pre-selected regions of interest (ROI) as is typical in both DCM and GCM. However, there has been recent interest in making *R* a continuous manifold (i.e. the cortex) that is approximated by a very high dimensional representation at the voxel level. We denote the complete set of random variables associated with each node as *X* = {*X*_\ *i*_, *X*_*i*_} whose joint distribution is described using a generative model. *X*_\ *i*_ is the set of nodes without node *i* and *p*(*x*) ≜ *p*(*X* = *x*).

### The observation equation

Any model always includes an explicit or implicit observation equation that generally varies with the imaging modality. This equation specifies how hidden (neuronal) states *x*_*r*_(*t*) produce observable data *y*_*q*_(*t*_*k*_): *q* ∈ *Q*. This is the sensor data sampled at discrete time points *t*_*k*_ = *k Δ*:

(1) $$y_{q}\left(t_{k}\right)=g\left(x_{r},t\right)+e\left(t_{k}\right):r\in R_{r},t\in \left[t_{k},t_{k-1}\right]$$

for *k* = 1 … *K*. It is important to note that observations at a given sensor *q* only reflect neural states from a subset of brain sites, modified by the function *g* over a time interval determined by the sampling period Δt and corrupted by instrumental noise *e*(*t*_*k*_). When the sampling period is not considered explicitly, the observations are denoted by *y*_*q*_(*k*). In most cases, this mapping does not need to be dynamic since there is no physical feedback from observed data to brain processes. In this special case, the observation equation reduces to an instantaneous transformation: $Y\left(t\right)=\tilde{g}\left(X\left(t\right)\right)$, where $\tilde{g}$ is derived from *g* and any retarded (past) hidden states have been absorbed in *X*(*t*) (e.g., to model hemodynamic convolutions).

A selected collection of observation equations used in Neuroimaging is provided in Table 1. The observation equation is sometimes simplified by assuming that observed data is a direct measurement of neural states (with negligible error). While this might be an acceptable assumption for invasive electrophysiological recordings, it is inappropriate in many other situations: for example, much of the activity in the brain is reflected in the EEG/MEG via the lead field with a resultant spatial smearing. For the BOLD signal, the C&C articles have discussed exhaustively the need to account for temporal smearing produced by the hemodynamic response function (HRF) when analyzing BOLD responses. This is important for fMRI because the sampling period is quite large with respect to the time course of neural events (we shall elaborate on this below).

**Table 1:** Observation equations. Examples of observation equations used for causal modeling of effective connectivity in the recent literature. Abbreviations: discrete (D), continuous (C), white noise (WN). Note for Models #5 and #6 the observation equation is considered as all the equations except for the (neural) state equations. Strictly speaking, the observer function is just the first equality (because the subsequent equations of motion are part of the state equation); however, we have presented the equations like this so that one can compare instantaneous observation equations that are a function of hidden states, convolution operators or a set of differential equations that take hidden neuronal states as their inputs.

	Model	Observation equation	Measurement	Space	Time	Equation type	Kind of stochastic process
1	None (Bressler and Seth, 2010)	*y*(*r*, *k*) = *x*(*r*, *k*)	EEG/fMRI	D	D	Identity	none
2	Added noise (Nalatore et al., 2007)	*y*(*r*, *k*) = *x*(*r*, *k*) + *e*(*r*, *t*)	fMRI	D	D	Linear regression	WN
3	Spatial smearing (Riera et al., 2006)	*y*(*q*, *t*) = ∫ _*r* ∈ *R*_*k*(*r*, *r*′)*x*(*r*′, *t*)*dr*′ + *e*(*r*, *t*)	EEG/MEG	D	C	Volterra integral equation with noise	none
4	Convolution with linear HRF (Glover, 1999)	$y\left(r,k\right)=\int_{-\infty }^{t=k\Delta }h(\tau )x\left(r,t-\tau \right)d\tau +e\left(r,k\right)$	fMRI	D	C	Temporal convolution	WN
5	Nonlinear HRF function (Friston et al., 2000)	$\begin{array}{l} y_{t}=V_{0}\left(a_{1}\left(1-q_{t}\right)-a_{2}\left(1-v_{t}\right)\right)\\ {\dot{v}}_{t}=\frac{1}{\tau_{0}}\left(f_{t}-v_{t}^{1/\alpha }\right)\\ {\dot{q}}_{t}=\frac{1}{\tau_{0}}\left(\frac{f_{t}\left(1-{\left(1-E_{0}\right)}^{1/f_{t}}\right)}{E_{0}}-\frac{q_{t}}{v_{t}^{1-1/\alpha }}\right) \end{array}$ ${\dot{s}}_{t}=\varepsilon u_{t}-\frac{1}{\tau_{s}}s_{t}-\frac{1}{\tau_{f}}\left(f_{t}-1\right)$ ${\dot{f}}_{t}=s_{t}$	fMRI	C	C	Nonlinear differential equation	none
6	Nonlinear HRF function (Valdes-Sosa et al., 2009a)	$\begin{array}{l} \left\{ \begin{array}{l} {\dot{g}}_{e}\left(t\right)=s_{e}\left(t\right)\\ {\dot{s}}_{e}\left(t\right)=\frac{a_{e}}{\tau_{e}}\left(u_{e}\left(t-\delta_{e}\right)-1\right)-\frac{2}{\tau_{e}}s_{e}\left(t\right)-\frac{1}{\tau_{e}{}^{2}}\left(g_{e}\left(t\right)-1\right) \end{array}\right.\\ \left\{ \begin{array}{l} {\dot{g}}_{i}\left(t\right)=s_{i}\left(t\right)\\ {\dot{s}}_{i}\left(t\right)=\frac{a_{i}}{\tau_{i}}\left(u_{i}\left(t-\delta_{i}\right)-1\right)-\frac{2}{\tau_{i}}s_{i}\left(t\right)-\frac{1}{\tau_{i}{}^{2}}\left(g_{i}\left(t\right)-1\right) \end{array}\right.\\ x=\frac{1}{1+e^{-c\left(g_{e}\left(t\right)-d\right)}}\\ \left\{ \begin{array}{l} \overset{˙˙}{f}\left(t\right)=s_{f}\left(t\right)\\ {\dot{s}}_{f}\left(t\right)=\varepsilon \left(u_{e}\left(t-\delta_{f}\right)-1\right)-\frac{s_{f}\left(t\right)}{\tau_{s}}-\frac{f\left(t\right)-1}{\tau_{f}} \end{array}\right.\\ m_{i}\left(t\right)=g_{i}\left(t\right)\text{,}\;m_{e}\left(t\right)=\frac{2-x}{2-x_{0}}g_{e}\left(t\right)\text{,}\;m\left(t\right)=\frac{\gamma m_{e}\left(t\right)+m_{i}\left(t\right)}{\gamma +1}\\ \left\{ \begin{array}{c} \dot{v}\left(t\right)=\frac{1}{\tau_{0}}\left(f\left(t\right)-f_{\mathrm{out}}\left(v,t\right)\right)\\ \dot{q}\left(t\right)=\frac{1}{\tau_{0}}\left(m\left(t\right)-f_{\mathrm{out}}\left(v,t\right)\frac{q\left(t\right)}{v\left(t\right)}\right),f_{\mathrm{out}}\left(v,t\right)=v^{\frac{1}{\alpha }} \end{array}\right.\\ y\left(t\right)=V_{0}\left(a_{1}\left(1-q\right)-a_{2}\left(1-v\right)\right) \end{array}$	EEG/fMRI	C	C	Nonlinear random differential algebraic equation	none

Instrumental or sensor noise can seriously affect the results of causal analyses. One simple approach to causal modeling is to take the observation equation out of the picture by inverting the observation equation (i.e., mapping from data to hidden states). The estimated states can then used for determining effective connectivity. This approach has been taken both for the EEG (Supp et al., 2007) and fMRI (David et al., 2008). However, this is suboptimal because it assumes that the causal modeling of hidden states is conditionally independent of the mapping from data. This is generally not the case (e.g., non-identifiability between observation and evolution processes described below). The optimal statistical procedure is to invert the complete generative model, including the observation and state equations modeling the evolution of hidden states. This properly accommodates conditional dependencies between parameters of the observer and state equations. A nice example of this is DCM for EEG and MEG, in which a SSM of coupled neuronal sources and a conventional electromagnetic forward model are inverted together. This means the parameters describing the spatial deployment of sources (e.g., dipole orientation and location) are optimized in relation to parameters controlling the effective connectivity among hidden sources. This sort of combined estimation has been described for simple noise models (Table 1-#2 by Nalatore et al. (2007)). For fMRI, DCM models the hemodynamic response with hidden physiological states like blood flow and volume and then uses a nonlinear observer function to generate BOLD responses (Table 2-#4). Early applications of GCM did not model the HRF but in recent years a number of papers have included explicit observation models in GCM (Ge et al., 2009, Havlicek et al., 2010), which have even incorporated the full nonlinear HRF model used in DCM (Havlicek et al., 2009, Havlicek et al., 2011).

### The state equation

The evolution of the neuronal states is specified by the dynamical equations:

(2) $$x_{r}\left(t\right)=f\left(x_{r^{\prime }\in R_{r\prime }}\left(\tau \right),u\left(\tau \right),\xi_{r^{\prime }\in R_{r^{\prime }}}\left(\tau \right)\right):\tau \in \left(t,t-t_{0}\right]\text{.}$$

This equation^3^ expresses, *x*_*r*_(*t*), the state vector of node *r* at time *t* as a function of:

• the states of nodes *x*_*r* '_(*τ*): *r*′ ∈ *R*_*r* '_ ⊆ *R*
• exogenous inputs, *u*(*τ*) and a
• stochastic process *ξ*_*r*′_(*τ*).

Note that the dependence of the current states at node *r* may be contingent on values of other variables from an arbitrary past from *t* − *t*_0_ to just before *t*. The time dependence of Eq. (2) is important because it allows to model feedback processes within the network.

Many specific forms have been proposed for Eq. (2); some examples are listed in Table 2, which is just a selection to illustrate different points discussed below. Some types of equations, to our knowledge, have not been yet used for the analysis of effective connectivity. Several general observations emerge from these examples:

*Discrete versus continuous time modeling*: The state equations for GCM have been for the most part discrete time recurrence models (Bressler and Seth, 2010). Those for DCM are based on continuous time models (differential equations) (Friston, 2009a). The latter have advantages in dealing with the problem of temporal aggregation and sub-sampling as we shall see below. In fact, DCM is distinguished from general SSM by the fact it is based on differential equations of one sort or another.
*Discrete versus continuous spatial modeling*: GCM has been applied to continuous space (neural fields) though limited to discrete time (Galka et al., 2004, Valdes-Sosa, 2004). DCM has mainly been developed for discrete-space (ROIs) and, as mentioned above, continuous time. State space models that are continuous in space and time have recently been looked at in the context of neural field equations (Daunizeau et al., 2009c, Galka et al., 2008).
*Type of equation*: GCM has been predominantly based on linear stochastic recurrence (autoregressive) models (Bressler and Seth, 2010). DCM on the other hand has popularized the use of deterministic ordinary differential equations (ODE). These range from simple bilinear forms for fMRI that accommodate interactions between the input and the state variables (Friston, 2009a) to complicated nonlinear equations describing the ensemble dynamics of neural mass models. In their most comprehensive form, these models can be formulated as Hierarchical Dynamical Models (HDM) (Friston, 2008a, Friston, 2008b). HDM uses nonlinear random differential equations and static nonlinearities, which can be deployed hierarchically to reproduce most known parametric models. However, as noted in the C&C, GCM is not limited to linear models. GCM mapping (Roebroeck et al., 2005) uses an (implicit) bilinear model, because the Autoregressive coefficients depend on the stimulus; this bilinearity is explicit in GCM on manifolds (Valdés-Sosa et al., 2005) GCM has also been extended to cover nonlinear state-equations (Freiwald et al., 1999, Marinazzo et al., 2010).
  The type of models used as state equations are very varied (and are sometimes equivalent). One can find (for discrete spatial nodes) recurrence equations, ordinary differential equations, and (for neural fields) differential-integral and partial differential equations. To underscore the variety of forms for effective connectivity, we note entry #8 in Table 2 which boasts a fractional differential operator! Fractional operators arise in the context of neural fields in more than one dimension; they result from the Fourier transform of a synaptic connection density that is a continuous function of physical distance. However, the ensuing fractional differential operators are usually replaced by ordinary (partial) differential operators, when numerically solving the neural wave propagation equation given in Table 2; see Bojak and Liley (2010) and Coombes et al. (2007) for the so-called ‘long wavelength approximation’.
  Among other things, it can be important to include time delays in the state equation; this is usually avoided when possible to keep the numerics simple (delay differential equations are infinite dimensional) and are generally considered unnecessary for fMRI. However, delays are crucial when modeling electromagnetic data, since they can have a profound effect on systems dynamics (Brandt et al., 2007). For example, delayed excitatory connections can have an inhibitory instantaneous effect. In fact starting with Jansen and Rit (1995) it has been common practice to include time delays. This can be implemented within the framework of ODEs; David et al. (2006) describe an ODE approximation to delayed differential equations in the context of DCM for EEG and MEG.
  An example of the potential richness of model structures is found in Valdes-Sosa et al. (2009a) in a neural field forward model for EEG/fMRI fusion, which includes anatomical connections and delays as algebraic constraints. This approach (of including algebraic constraints) affords the possibility of building complex models from nonlinear components, using simple interconnection rules—something that has been developed for control theory (Shampine and Gahinet, 2006). Note that algebraic constraints may be added to any of the aforementioned forms of state equation.
*Type of stochastics*: for GCM-type modeling with discrete-time models, Gaussian White Noise (GWN) is usually assumed for the random fluctuations (state noise) or driving forces (innovations) for the SSM and poses no special difficulties. However in continuous time the problem becomes more intricate. A popular approach is to treat the innovation as nowhere differentiable but continuous Gaussian White Noise (the “derivative” of Brownian motion (i.e., a Wiener process). When added to ordinary differential equations we obtain “stochastic differential equations” (SDE) as described in Medvegyev (2007) and used for connectivity analysis of neural masses in Riera et al., 2007a, Riera and al., 2007b, Riera et al., 2006. Wiener noise is also central to the theory of Stochastic Partial Differential Equations (SPDE) (Holden et al., 1996), which may play a similar role in neural field theory as SDEs have played for neural masses (Shardlow, 2003).
  Despite the historical predominance of the classical SDE formulation in econometrics (and SSM generally), we wish to emphasize the following developments, which may take us (in the biological sciences) in a different direction:
  1. The first is the development of a theory for “random differential equations” (RDE) (Jentzen and Kloeden, 2009). Here randomness is not limited to additive Gaussian white noise because the parameters of the state equations are treated as stochastic. RDE are treated as deterministic ODE, in the spirit of Sussmann (1977), an approach usable to great advantage in extensive neural mass modeling (Valdes-Sosa et al., 2009a) that is implicitly a neural field.
  2. The second development, also motivated by dissatisfaction with classical SDE was introduced in Friston and Daunizeau (2008). In that paper, it was argued that DCMs should be based on stochastic processes, whose sample paths are infinitely differentiable—in other words, analytic and non-Markovian. Though overlooked in the heyday of SDE theory, this type of process was described very on early by Belyaev (1959).^4^ In fact any band-limited stochastic process is an example of an analytic random process; a stochastic process with a spectrum that decreases sharply with frequency, has long memory, and is non-Markovian (Łuczka, 2005). The connection between analytic stochastic processes and RDE can be found in Calbo et al. (2010). An interesting point here is that for the process to be analytic its successive derivatives must have finite variances, as explained in Friston and Daunizeau (2008). This leads to the generalization of classical SSM into generalized coordinates of motion that model high-order temporal derivatives explicitly. As pointed out in Friston, 2008a, Friston, 2008b, it is possible to cast an RDE as a SDE by truncating the temporal derivatives at some suitably high order (see also Carbonell et al., 2007). However, this is not necessary because the theory and numerics for RDEs in generalized coordinates are simpler than for the equivalent SDE (and avoid the unwieldy calculus of Markovian formulations, due to Ito and Stratonovich).
  3. The third development is the recognition that non-Markovian processes may be essential for neurobiological modeling. This has been studied for some time in physics (Łuczka, 2005) but has only recently been pointed out by Friston, 2008a, Friston, 2008b in a neuroscience setting. In fact, Faugeras et al. (2009) provide a constructive mean-field analysis of multi-population neural networks with random synaptic weights and stochastic inputs that exhibits, as a main characteristic, the emergence of non-Markovian stochastics.
  4. Finally the fourth development is the emergence of neural field models (Coombes, 2010, Deco et al., 2008), which not only poses much larger scale problems but also the use of integral equations, differential–integral equations, and partial differential equations which have yet to be exploited by DCM or GCM.
*Biophysical versus non-parametric motivation*: As discussed above, there is an ever increasing use of biophysically motivated neural mass and field state equations and, in principle, these are preferred when possible because they bring biophysical constraints to bear on model inversion and inference. When carrying out exploratory analyses with very large SSM, it may be acceptable to use simple linear or bilinear models as long as basic aspects of modeling are not omitted.
*Further generalizations*: We want to end this subsection by mentioning that there is a wealth of theory and numerics for other stochastic (point) processes (Aalen and Frigessi, 2007, Commenges and Gégout-Petit, 2009) that have not yet been, to our knowledge, treated formally in Neuroimaging. Spike trains, interictal-spikes, and random short-timed external stimuli may be treated as point processes and can be analyzed in a unified framework with the more familiar continuous time series. This theory even encompasses mixtures of slow wave and spike trains.
  Causal modeling depends very specifically on the temporal and spatial scales chosen and the implicit level of granularity chosen to characterize functional brain architectures. For example, if we were to study the interaction of two neural masses and model the propagation of activity between them in detail, we would have to make use of the PDE that describes the propagation of nerve impulses. If we eschew this level of detail, we may just model the fact that afferent activity arrives at a neural mass with a conduction delay and use delay differential equations. In short, the specification of the appropriate SSM depends on the spatial and temporal scale that one is analyzing. For example, in concurrent EEG/fMRI analysis of resting state oscillations (Martínez-Montes et al., 2004) the temporal scale of interesting phenomena (fluctuations of the EEG spectrum) is such that one may *convolve* the EEG signal and do away with the observation equation! This is exactly the opposite of the deconvolution approach mentioned above. The purpose of Table 1, Table 2 is to highlight the variety of forms that both state and observation equations can take; for example, in Table 2-#6 key differential equations are transformed into differential algebraic equations to great computational advantage (Valdes-Sosa et al., 2009a).

**Table 2:** State equations. Examples of the state equations used in the recent literature for causal modeling of effective connectivity. Abbreviations: C (continuous), D (discrete), WN (white noise).

	Model	State equation	Space	Time	Equation type	Stochastic process
	Linear GCM (Bressler and Seth, 2010)	$x\left(r,k\right)=\sum_{r'=1}^{N_{r}}\sum_{l=1}^{T}a_{l}(r,r^{\prime })x\left(r^{\prime },k-l\right)+\xi \left(r,k\right)$	D	D	Linear multivariate linear autoregressive (VAR)	WN
2	Nonlinear GCM (Freiwald et al., 1999)	$x\left(r,k\right)=\sum_{r'=1}^{N_{r}}\sum_{l=1}^{T}a\left[l,r,r^{\prime };x\left(r^{\prime },k-l\right)\right]\;\;x\left(r^{\prime },k-l\right)+\xi \left(r,k\right)$	D	D	Nonlinear nonparametric VAR (NNp_MVAR)	WN
3	Linear bivariate GCM mapping (Roebroeck et al., 2005)	$\begin{array}{l} \left[\begin{array}{c} x\left(r,k\right)\\ x\left(ROI,k\right) \end{array}\right]=\sum_{l=1}^{Nl}\left[\begin{array}{cc} a_{l}(r,r) & a_{l}(r,ROI)\\ a_{l}(ROI,r) & a_{l}(ROI,ROI) \end{array}\right]\left[\begin{array}{c} x\left(r,k-l\right)\\ x\left(ROI,k-l\right) \end{array}\right]+\left[\begin{array}{c} \xi \left(r,k\right)\\ \xi \left(ROI,k\right) \end{array}\right]\;\\ \forall r\in R\;x\left(ROI,k\right)=\int_{r\in R}x\left(r,k\right)\;dr \end{array}$	D	D	VAR since *a*_*l*_(*r*, *r*') Implicitly bi-linear changes with state. (GCMap)	WN
4	Linear GCM on spatial manifold (Valdés et al., 2006)	$x\left(r,k\right)=\sum_{l=1}^{Nl}\int_{r'\in R}a_{l}(r,r^{\prime })x\left(r^{\prime },k-l\right)dr^{\prime }+\xi \left(r,k\right)$	C	D	Implicitly bi-linear VAR as in 3	WN
5	Nonlinear DCM (Stephan et al., 2008)	$\begin{array}{c} \dot{x}\left(r,t\right)=\sum_{r'=1}^{Nx}a\left(r,r^{\prime }\right)\;x\left(r^{\prime },t\right)\\ +\sum_{i=1}^{Nu}u\left(i,t\right)\sum_{r'=1}^{Nx}b\left(r,r^{\prime }\right)\;x\left(r^{\prime },t\right)\;\\ +\sum_{r'=1}^{Nx}\sum_{r''=1}^{Nx}d\left(r,r^{\prime },r^{\prime \prime }\right)\;x\left(r^{\prime },t\right)\;x\left(r^{\prime \prime },t\right)+\sum_{i=1}^{Nu}c\left(r,i\right)\;u\left(i,t\right) \end{array}$	D	D	Differential equation bilinear in both states and inputs (DE)	None
6	Neural mass model (Valdes et al., 1999)	$\dot{x}\left(r,t\right)=f\left(x(r,t)\right)+\xi \left(r,t\right)$	C	C	Ito stochastic differential (SDE)	WN as formal derivative of Brownian motion
7	Hierarchical dynamic causal model (Friston, 2008a, Friston, 2008b)	$\dot{x}\left(r,t\right)=f\left(x(r,t),u\left(t\right)\right)+\xi \left(r,t\right)$	D	C	General nonlinear (HDM)	Analytic, non-Markovian
8	Neural field (Jirsa et al., 2002)	${\left(\frac{\partial^{2}}{\partial t^{2}}+2\omega \frac{\partial }{\partial t}+\omega_{0}^{2}-v^{2}\nabla^{2}\right)}^{3/2}x(r,t)=\left(\omega_{0}^{3}+\omega_{0}^{2}\frac{\partial }{\partial t}\right)\;S\left[x(r,t)+\xi \left(r,t\right)\right]$	C	C	Stochastic fractional partial differential (SfPDE)	WN
9	Modified neural field (P. A. Valdes-Sosa et al., 2009a)	$\begin{array}{l} \ddot{x}\left(r,t\right)=f\left(\dot{x}\left(r,t\right),x\left(r,t\right)\right)+S\left(z\left(r,t\right)\right)+\xi \left(r,t\right)\\ z\left(r,t\right)=\int_{R}a\left(r,r'\right)\;x\left(r,\tau (r,r')\right)\;dr'\\ \tau (r,r')=t-\frac{\left|r-r'\right|}{\nu } \end{array}$	C	C	Random differential–algebraic-equation (RDE)	General

### Specification of priors

It is safe to say that the Neuroimaging (and perhaps generally) modeling can be cast as Bayesian inference. This is just a euphemism for saying that inference rests on probability theory. The two key aspects of Bayesian inference we will appeal to in this article are (i) the importance of prior believes that form an explicit part of the generative model; and (ii) the central role of Bayesian model evidence in optimizing (comparing and selecting) models to test hypotheses. In terms of priors, it was very clear in an early state space model for EEG connectivity (Valdes-Sosa et al., 1996) that without prior assumptions about the spatial and temporal properties of the EEG, it was not possible to even attempt source reconstruction. Indeed the whole literature on ill-posed inverse problems rests on regularization that can be cast in terms of prior beliefs.

In the SSM formulation, priors may be placed upon parameters in the observation and state equations, and the states themselves (e.g., through priors on the higher-order motion of states or state-noise). Sometimes, it may be necessary to place priors on the priors (hyperpriors) to control model complexity. There has been an increasing use of priors in fMRI research, as clearly formulated in the DCM and HDM framework (Friston, 2008a, Friston, 2008b). In connectivity analyses, in addition to the usual use of priors to constrain the range of parameters quantitatively; formal or structural priors are crucial for switching off subsets of connections to form different (alternative) models of observed data. Effectively, this specifies the model in terms of its adjacency matrix, which defines allowable connections or conditional dependencies among nodes. Conditional independence (absence of an edge or anti-edge) is easy to specify by using a prior expectation of zero and with zero variance. This is an explicit part of model specification in DCM and is implicit in Granger tests of autoregressive models, with and without a particular autoregression coefficient.

Crucially, formal priors are not restricted to the parameters of a model; they can also be applied to the form of the prior density over parameters. These can be regarded as formal hyperpriors. An important example here is the prior belief that connections are distributed sparsely (with lots of small or absent connections and a small number of strong connections). This sort of hyperprior can be implemented by assuming the prior over parameters is sparse. A nice example of this can be found in Valdés-Sosa (2004), Valdés-Sosa et al. (2005, 2006), and Sánchez-Bornot et al. (2008).

**Fig. 3:**
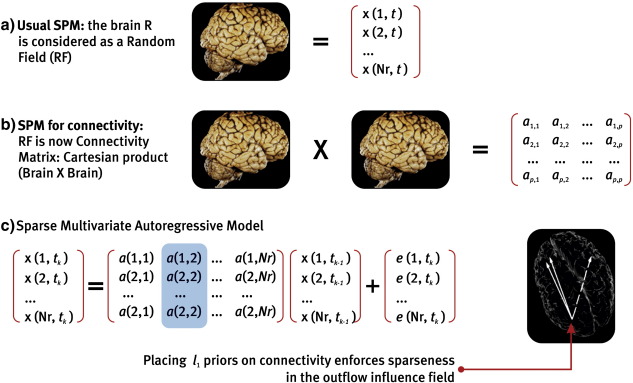
Bayesian inference on the connectivity matrix as a random field. a) Causal modeling in Neuroimaging has concentrated on inference on neural states *x*(*r*, *t*) ∈ *R* defined on a subset of nodes in the brain. However, spatial priors can be used to extend models into the spatial domain (cf., minimum norm priors over current source densities in EEG/MEG inverse problems). b) In connectivity analysis, attention shifts to the AR (connectivity) matrix (or function) *a*(*r*, *r*′), where the ordered pairs (*r*, *r*′) belong to the Cartesian product *R* × *R*. For this type of inference, priors are now placed on the connectivity matrix. c) Sparse multivariate autoregression obtains by penalizing the columns of a full multivariate autoregressive model (Valdés-Sosa et al., 2005) thus forcing the columns of the connectivity matrix to be sparse. The columns of the connectivity matrix are the “outfields” that map each voxel to the rest of the brain. This is an example of using sparse (spatial) hyperpriors to regularize a very difficult inverse problem in causal modeling.

**Fig. 4:**
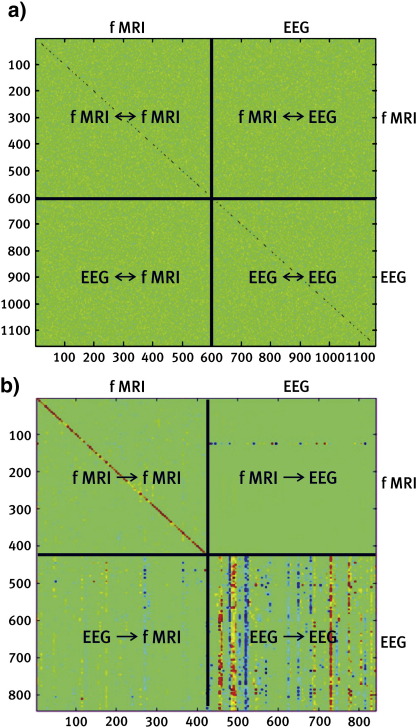
Sparse multivariate autoregression of concurrent EEG/fMRI recordings. Intra and inter modality connectivity matrix for a concurrent EEG/fMRI recordings. The data analyzed here were the time courses of the average activity in 579 ROI: for BOLD (first half of data vector) and EEG power at the alpha peak. A first-order sparse multivariate autoregressive model was fitted with an *l*_1_ norm (hyper) prior on the coefficient matrix. The t-statistics of the autoregression coefficients where used for display. The color bar is scaled to the largest absolute value of the matrix, where green codes for zero. a) the innovation covariance matrix reflecting the absence of contemporaneous influences: b) t-statistics for the lag 1 AR coefficients.

The essential features of their model are shown in Fig. 3. The authors analyzed slow fluctuations in resting state EEG. In this situation, convolving these electrophysiological fluctuations with a HRF affords (convolved) EEG and BOLD signals on the same time scale, permitting lag-based inference. An example is presented in Fig. 4, which shows the results of GCM Mapping for 579 ROIs from an EEG inverse solution and concurrent BOLD signals. The EEG sources were obtained via a time resolved VARETA inverse solution (Bosch-Bayard et al., 2001) at the peak of the alpha rhythm. The graphs present the result of inverting a (first order) multivariate vector autoregression model, where a sparse *l*_1_ norm penalty was imposed on the parameters (coefficient matrix). The implications of these results will be further discussed in Conclusion and suggestions for further work section below.

### Model comparison and Identifiability

As we have seen, the SSMs considered for EEG and fMRI analysis are becoming increasingly complex, with greater spatial or temporal coverage and improved biological realism. A fundamental question arises: *Are these models identifiable?* That is to say, are all states and parameters uniquely determined by a given set of data? This is a basic issue for all inverse problems, and indeed we are faced with a dynamical inverse problem of the greatest importance. For example, recent discussions about whether lag information can be derived from the fMRI signal (in spite of heavy smoothing by the HRF and the subsequent sub sampling) can be understood in terms of the identifiability of delays in the corresponding SSM. It is striking that, in spite of much classical work on the Identifiability of SSMs (see for example Ljung and Glad, 1994), a systematic treatment of identification has not been performed for Neuroimaging models (but see below). An example of the type of problem encountered is the complaint that a model with many neural masses and different configurations or parameter values can produce traces that “look the same as an observed response”.

Identifiability has been addressed in bioinformatics, where much theory for nonlinear SSM has been developed (Anguelova and Wennberg, 2010, August and Papachristodoulou, 2009). Of particular note is DAISY, a computer algebra system for checking nonlinear SSM Identifiability (Saccomani et al., 2010). Another framework for modeling and fitting systems defined by differential equations in bioinformatics is “Potters Wheel” (Maiwald and Timmer, 2008), which uses a profile likelihood approach (Raue et al., 2009) to explore “practical Identifiability” in addition to structural (theoretical) Identifiability. So why has Neuroimaging not developed similar schemes?

In fact, it has. In a Bayesian setting the issue of model (and parameter) identifiability is resolved though Bayesian model comparison. If two models generate exactly the same data with the same number of parameters (complexity), then their evidence will be identical. This means there is no evidence for one model over the other and they cannot be distinguished. We will refer a lot to model evidence in what follows: model evidence is simply the probability of the data given the model. It is the marginal likelihood that obtains from marginalizing the likelihood over unknown model parameters. This is useful to remember because it means the likelihood of a model (the probability of data given a model and its parameters) is a special case of model evidence that results when we ignore uncertainty about the parameters. In the same way, classical likelihood ratio tests of two models are special cases of Bayes Factors used in Bayesian model comparison. In this context, identifiability is a particular aspect of model comparison. Identifiability mandates that changing a component of a model changes the model evidence. This is the basic idea behind the profile likelihood approach (Raue et al., 2009), which is based on the profile of the evidence for models with different parameter values. There are other examples that can be regarded as special cases of model comparison; for example, the Kullback–Leibler information criterion proposed for model identification (Chen et al., 2009). The evidence can be decomposed into an accuracy and complexity term (see Penny et al., 2004). Interestingly, the complexity term is the Kullback–Leibler divergence between the posterior and prior densities over parameters. This means that in the absence of informative priors, model evidence reduces to accuracy; and identifiability reduces to a (nontrivial) change in the accuracy or fit when changing a model or parameter.

The Bayes–Net literature (see below) has dealt with the problem of Identifiability for graphical causal models at its inception (Spirtes et al., 2000). It can be shown that a given data set can be compatible not with a single causal model but with an equivalence class of models (that all have the same evidence). The implications of this for Neuroimaging have been considered in Ramsey et al. (2010). From this discussion, it becomes clear that the ability to measure model evidence (or some proxy) is absolutely essential to make sensible inferences about models or architectures generating observed data. This is at the heart of evidence-based inference and DCM.

### Summary

State space models for Neuroimaging come in an ever increasing variety of forms (Table 1, Table 2). It is useful to classify the types of models used in terms of their observation and state equations, as in Table 3. Here, we see a distinction between models that are fairly generic (in that they are not based on biophysical assumptions) and those that correspond to biologically informed models. The canonical HRF model is an example of generic HRF. Conventional GCM is based on a generic model for neural states: the VAR model and has been extended to switching VAR and bilinear models, the latter used in some forms of DCM. Being generic is, at the same time, a strength and weakness; biophysical models allow much more precise and informed inference—but only if the model is right or can be optimized in terms of its evidence. We have also seen the key role that model evidence plays in both making causal inferences by comparing models and (implicitly) establishing their identifiability. The evidence for a model depends on both accuracy and complexity and the complexity of the model depends on its priors.

**Table 3:** Classification of observation and state equations used in Neuroimaging state-space models. Generic models lack specific biophysical constraints but are widely applicable. Biophysically informed models are hypothesis driven and may afford more efficient inference (if correct). The term parametric refers to models with a small enough parameter set to be identifiable without additional priors but that may yield biased estimators. Nonparametric models are richly parameterized and therefore require prior distributions to be estimable but are generally unbiased.

	Observation model		State model	
	Parametric	Non-parametric	Parametric	Non-parametric
Generic	Linear canonical HRF (Glover, 1999)	Linear spline HRF (Marrelec et al., 2003)	GCM (Bressler and Seth, 2010) Switching VAR (Smith et al., 2010a) bilinear discrete DCM (Penny et al., 2005)	GCMap (Roebroeck et al., 2005)
Biophysically informed	DCM nonlinear HRF (Friston et al., 2000)	–	Neural mass models (Valdes et al., 1999) Biophysical DCM (Moran et al., 2008)	Neural fields (Daunizeau et al., 2009c)

Another distinction between models is their complexity (e.g., number of parameters they call on). It is clear that without prior beliefs, one cannot estimate more parameters than the degrees of freedom in the data available. However, modern statistical learning has gone beyond low dimensional parametric models to embrace non-parametric models with very high dimensional parameter spaces. The effective number of degrees of freedom is controlled by the use of priors. DCM has been concerned mainly with hypothesis driven parametric models, as has conventional GCM. However, nonparametric models, such as smoothness priors in the time domain have been used to estimate the HRF (Marrelec et al., 2003). Another example is the use of spatial priors to estimate the connectivity matrix in GCMap (Valdes-Sosa, 2004). Finally, when choosing a State Space model, it is useful to appreciate that there are two agendas when trying to understanding the connectivity of complex systems:

1. A data driven exploratory (discovery) approach that tries to scan the largest model space possible, identifying robust phenomena or candidates that will serve as constraints for more detailed modeling. This type of approach generally uses nonparametric or simply parameterized models for knowledge discovery. Prior knowledge is generally nonspecific (e.g., connections are sparse) but relatively non-restrictive.
2. A model driven confirmatory approach that is based on specific hypothesis driven models that incorporate as much biophysical prior knowledge as possible. Generally, the priors entail specific hypothesis about connectivity that can be resolved using model comparison.

These two approaches are shown in Fig. 2 (modified from Valdés-Sosa et al., 1999). In both cases, modeling is constrained by the data, by biophysical plausibility and ultimately the ability to establish links with computational models (hypotheses) of information processing in the brain. Table 3 shows that at one extreme the model-driven approach is epitomized by Generic Nonparametric Models. Here, modeling efforts are constrained by data and the attempt to disclose emergent behavior, attractors and bifurcations (Breakspear et al., 2006) that can be checked against biophysically motivated models. An example of this approach is searching the complete brain times brain connectivity space (Fig. 3) with GCM mapping (Valdes-Sosa, 2004, Roebroeck et al., 2005). At the other end we have the parametric and biophysically informed approach that DCM has emphasized (Chen et al., 2008). Having said this, as evidenced by this paper and companion papers, there is convergence of the two approaches, with a gradual blurring of the boundaries between DCM and GCM.

## Model inversions and inference

In this section, we look at the problem of model identification or inversion; namely, estimating the states and parameters of a particular model. It can be confusing when there is discussion of a new model that claims to be different from previous models, when it is actually the same model but with a different inversion or estimation scheme. We will try to clarify the distinction between models and highlight their points of contact when possible. Our main focus here will be on different formulations of SSM and how these formulations affect model inversion.

### Discrete or continuous time?

One (almost) always works with discretely sampled data. When the model is itself discrete, then the only issue is matching the sampling times of the model predictions and the data predicted. However, when starting from a continuous time model, one has to model explicitly the mapping to discrete time.

Mapping continuous time predictions to discrete samples is a well-known topic in engineering and (probably from the early 50s) has been solved by linearization of the ODEs and integration over discrete time steps; a method known as the Exponential Euler method for reasons we shall see below: see Minchev and Wright (2005) for a historical review. For a recent review, with pointers to engineering toolboxes, see Garnier and Wang (2008).

One of the most exciting developments in the 60s, in econometrics was the development of explicit methods for estimating continuous models from sampled data, initiated by Bergstrom (1966).^5^ His idea was essentially the following. Consider 3 time series *X*_1_(*t*), X_2_(*t*), and *X*_3_(*t*) where we know the values at time *t*:

(3) $$\left(\begin{array}{l} dX_{1}\left(t\right)\\ d{\text{X}}_{2}\left(t\right)\\ d{\text{X}}_{3}\left(t\right) \end{array}\right)=A\left[\begin{array}{l} X_{1}\left(t\right)\\ X_{2}\left(t\right)\\ X_{3}\left(t\right) \end{array}\right]dt+\sum^{1/2}dB\left(t\right)\text{.}$$

Then the explicit integration^6^ over the interval $\left[t+\Delta t,t\right]$ is

(4) $$\begin{array}{c} \left(\begin{array}{l} X_{1}\left(t+\Delta t\right)\\ X_{2}\left(t+\Delta t\right)\\ X_{3}\left(t+\Delta t\right) \end{array}\right)=\text{exp}\left(A\Delta t\right)\left(\begin{array}{l} X_{1}\left(t\right)\\ X_{2}\left(t\right)\\ X_{3}\left(t\right) \end{array}\right)+e\left(t+\Delta t\right)\\ e\left(t+\Delta t\right)=\int_{0}^{\mathrm{\Delta t}}\text{exp}\left(sA\right)\sum^{1/2}dB\left(t-s\right)\\ \Sigma_{\mathrm{discrete}}=\int_{0}^{\mathrm{\Delta t}}\text{exp}\left(sA\right)\sum \text{exp}\left(sA^{T}\right)ds\\ e\left(t+\Delta t\right)\sim N(0,\Sigma_{\mathrm{discrete}})\text{.} \end{array}$$

The noise of the discrete process now has the covariance matrix *Σ*_*discrete*_. It is immediately evident from the equation above that the lag zero covariance matrix *Σ*_*discrete*_ will show contemporaneous covariance even if the continuous covariance matrix *Σ* is diagonal. In other words, the discrete noise becomes correlated over the three time-series (e.g., channels). This is because the random fluctuations ‘persist’ through their influence on the motion of the states. Rather than considering this a disadvantage Bergstrom, 1984, Phillips, 1974 initiated a line of work studying the estimation of continuous time Autoregressive models (Mccrorie and Chambers, 2006), and continuous time Autoregressive Moving Average Models (Chambers and Thornton, 2009). This approach tries to use both lag information (the AR part) and zero-lag covariance information to identify the underlying linear model.

The extension of the above methods to nonlinear stochastic systems was proposed by Ozaki (1992) and has been extensively developed in recent years, as reviewed in Valdes-Sosa et al. (2009a). Consider a nonlinear system of the form:

(5) $$\begin{array}{c} dX\left(t\right)=f\left(X(t)\right)dt+\sum^{1/2}dB\left(t\right)\\ X\left(t\right)=\left[\begin{array}{l} X_{1}\left(t\right)\\ X_{2}\left(t\right)\\ X_{3}\left(t\right) \end{array}\right]\text{.} \end{array}$$

The essential assumption in local linearization (LL) of this nonlinear system is to consider the Jacobian matrix *A* = ∂ *f*/∂ *X* as constant over the time period, $\left[t+\Delta t,t\right]$. This Jacobian plays the same role as the matrix of autoregression coefficient in the linear systems above. Integration over this interval follows as above, with the solution:

(6) $$X\left(t+\Delta t\right)=X\left(t\right)+A^{-1}(\exp\left(A\Delta t\right)-I)f\left(X\left(t\right)\right)+e\left(t+\Delta t\right)$$

^7^where *I* is the identity matrix. This is solution is locally linear but crucially it changes with the state at the beginning of each integration interval; this is how is accommodates nonlinearity (i.e., a state-dependent autoregression matrix). As above, the discretised noise shows instantaneous correlations. Examples of inverting nonlinear continuous time neural models using this procedure are described in Valdes-Sosa et al., 1999, Riera and al., 2007b, Friston and Daunizeau, 2008, Marreiros et al., 2009, Stephan et al., 2008, and Daunizeau et al. (2009b). Local linearization of this sort is used in all DCMs, including those formulated in generalized coordinates of motion.

There are several well-known technical issues regarding continuous model inversion:

1. The econometrics literature has been very much concerned with identifiability in continuous time models—an issue raised by one of us in the C&C series (Friston, 2009b) due to the non-uniqueness of the inverse mapping of the matrix exponential operator(matrix logarithm) for large sampling periods Δt. This is not a problem for DCM, which parameterizes the state-equation directly in terms of the connectivity *A*. However, autoregressive models (AR) try to estimate $A=\exp\left(A\Delta t\right)$ directly, which requires a mapping $A=\frac{1}{\Delta t}\text{ln}\left(A\right)$ to get back to the underlying connectivity. Phillips noted in the 70s that A is not necessarily invertible, unless one is sampling at twice the highest frequency of the underlying signal (the Nyquist frequency) (Phillips, 1973); in other words, unless one samples quickly, in relation to the fluctuations in hidden states. In econometrics, there are several papers that study the conditions in which under-sampled systems can avoid an implicit aliasing problem (Hansen and Sargent, 1983, Mccrorie and Chambers, 2006, Mccrorie, 2003). This is not a problem for electrophysiological models because sampling is fast relative to the underlying neuronal dynamics. However, for fMRI this is not the case and AR models provide connectivity estimates, $A=\frac{1}{\Delta t}\ln\left(A\right)\in C^{N\times N}$ that are not necessarily unique (a phenomenon known as “aliasing” as discussed below).We will return to this problem in the next section, when considering the mediation of local (direct) and global (indirect) influences over time. Although this “missing time” problem precludes inference about coupling between neuronal states that fluctuate quickly in relation to hemodynamics, one can use AR models to make inferences about slow neuronal fluctuations based on fMRI (e.g., the amplitude modulation of certain frequencies; see Fig. 4). Optimal sampling for AR models has been studied extensively in the engineering literature—the essential point being that sampling should not be below or even much above the optimal choice that matches the natural frequencies (time-constants) of the hidden states (Astrom, 1969, Larsson et al., 2006).
2. When the sampling period Δt is sufficiently small, the AR model is approximately true. What is small? We found very few practical recommendations, with the exception of Sargan (1974), who uses heuristic arguments and Taylor expansions to suggest that a sampling frequency 1.5 times faster than the Nyquist frequency allows the use of a bilinear (or Tustin) approximation in (two stage non-recursive) autoregression procedures. As shown in the references cited above, it might be necessary to sample at several times the Nyquist frequency to use AR models directly. However, an interesting “Catch 22” emerges for AR models: The aliasing problem mandates fast sampling, but fast sampling violates Markovian (e.g., Gaussian noise) assumptions, if the true innovations are real (analytic) fluctuations.
3. A different (and a more complicated) issue concerns the identifiability of models of neural activity actually occurring at rates much higher than the sampling rates of fMRI, even when a DCM is parameterized in terms of neuronal coupling. This is an inverse problem that depends on prior assumptions. There are lessons to be learned from the EEG literature here: Linear deconvolution methods for inferring neural activity from EEG proposed by Glover, 1999, Valdes-Sosa et al., 2009a correspond to a temporal version of the minimum norm and LORETA spatial inverse solutions respectively. Riera et al. (2007a) and Riera et al. (2006), proposed a nonlinear deconvolution method. In fact, every standard SPM analysis of fMRI data is effectively a deconvolution, where the stimulus function (that is convolved with an assumed HRF) provides a generative model whose inversion corresponds to deconvolution. In the present context, the stimulus function provides the prior expectations about neuronal activity and the assumed HRF places priors on the ensuing hemodynamics. In short, model inversion or deconvolution depends on priors. The extent to which identifiability will limit inferences about neuronal coupling rests on whether the data supports evidence for different models of neuronal activity. We already know that there is sufficient information in fMRI time series to resolve DCMs with different neuronal connectivity architectures (through Bayesian model comparison), provided we use simple bilinear models. The issue here is whether we can make these models more realistic (cf., the neural mass models used for EEG) and still adjudicate among them, using model evidences: When models are too complex for their data, their evidence falls and model selection (identification) fails. This is an unresolved issue.

As one can see from these points, the issue of inference from discretised data depends on the fundamental frequencies of fluctuations in hidden states, data sampling rate, the model, and *the prior information we bring to the inferential problem*. When writing these lines, we were reminded of the dictum, prevalent in the first years of EEG source modeling, that one could “only estimate a number of dipoles that was less than or equal to a sixth of the number of electrodes”. Bayesian modeling has not increased the amount of information in data but it has given us a principled framework to optimize generative or forward models (i.e., priors) in terms of their complexity, by choosing priors that maximize model evidence. This has enabled advances in distributed source modeling and the elaboration of better constraints (Valdés-Sosa et al., 2009b). One might anticipate the same advances in causal modeling over the next few years.

### Time, frequency or generalized coordinates?

A last point to mention is that (prior to model inversion) it may be convenient to transform the time domain data to a different coordinate system, to facilitate computations or achieve a theoretical objective. In particular transformation to the frequency domain has proved quite useful.

1. This was proposed first for generic linear models in both continuous and discrete time (Robinson, 1991). More recently a nonparametric frequency domain approach has been proposed for Granger Causality (Dhamala et al., 2008).
2. A recent stream of EEG/MEG effective connectivity modeling has been introduced by Nolte et al., 2008, Marzetti et al., 2008, Nolte et al., 2009, andNolte et al. (2006) with the realization that time (phase) delays are reflected in the imaginary part of the EEG/MEG cross-spectra, whereas the real part contains contemporaneous contributions due to volume conduction.
3. Linearised versions of nonlinear DCMs have also been transformed successfully to the frequency domain (Moran et al., 2008, Robinson et al., 2008).

As noted above Friston, 2008a, Friston, 2008b has proposed a transformation to generalized coordinates, inspired by their success in physics. This involves representing the motion of the system by means of an infinite sequence of derivatives. The truncation of this sequence provides a summary of the time-series, in much the same way that a Fourier transform provides a series of Fourier coefficients. In classical time series analysis, the truncation is based on frequencies of interest. In generalized coordinates, the truncation is based on the smoothness of the time series. This use of generalized coordinates in causal modeling is predicated on the assumption that real stochastic processes are analytic (Belyaev, 1959).

### Model inversion and inference

There are many inversion schemes to estimate the states, parameters and hyperparameters of a model. Some of the most commonly used are variants of the Kalman Filer, Monte-Carlo methods and variational methods (se e.g., Daunizeau et al., 2009b for a variational Bayesian scheme). As reviewed in Valdes-Sosa et al. (2009a) the main challenges are how to scale the numerics of these schemes for more realistic and extensive modeling. The one thing all these schemes have in common is that they (implicitly or explicitly) optimize model parameters with respect to model evidence. In this sense model inversion and inference on models *per se* share a common objective; namely to maximize the evidence for a model.

Selecting or optimizing a model for effective connectivity ultimately rests on model evidence used in model comparison or averaging. The familiar tests for GCM (i.e. Dickey–Fuller test) are based on likelihood comparisons. As noted above, the likelihood (the probability of the data given a model and its parameters) is the same as model evidence (the probability of the data given a model), if we ignore uncertainty about the model parameters. However, the models considered in this paper, that include qualitative prior beliefs call for measures of goodness that balance accuracy (expected log-likelihood) with model complexity. All of these measures (AIC, BIC, GCV, and variational free energy) are approximations to the model evidence (Friston, 2008a, Friston, 2008b). Model evidence furnishes the measure used for the final probabilistic inference about a causal architecture (i.e., causal inference). Clearly, to carry out model comparison one must have an adequate set of candidates. Model Diagnostics are useful heuristics in this context that ensure that the correct models have been chosen for comparison. An interesting example that can be used to perform a detailed check of the adequacy of models is to assess the spatial and temporal whiteness of the residual innovation of the model which is illustrated in (Galka et al., 2004). More generally, the specification and exploration of model sets (spaces) probably represents one of the greatest challenges that lie ahead in this area.

### Summary

In summary, we have reviewed the distinction between autoregression (AR) models and models formulated in continuous time (DCM). We have touched upon the important role of local linearisation in mapping from continuous dynamics of hidden states to discrete data samples and the implications for sampling under AR models. In terms of model inversion and selection, we have highlighted the underlying role played by model evidence and have cast most of the cores issues in model identifiability and selection in terms of Bayesian model comparison. This subsumes questions about the complexity of models that can be supported by fMRI data; through to ultimate inferences about causality, in terms of which causal model has the greatest evidence. This section concludes our review of pragmatic issues and advances in the causal modeling of effective connectivity. We now turn to more conceptual issues and try to link the causal modeling for Neuroimaging described in this section to classical constructs that have dominated the theoretical literature over the past few decades.

## Statistical causal modeling

In this section, we review some key approaches to statistical causality. At one level, these approaches have had relatively little impact on recent developments in causal modeling in Neuroimaging, largely because they based on classical Markovian (and linear) models or ignore dynamics completely. However, this field contains some deep ideas and we include this section in the hope that it will illuminate some of the outstanding problems we face when modeling brain connectivity. Furthermore, it may be the case that bringing together classical temporal precedence treatments with structural causal modeling will finesse these problems and inspire theoreticians to tackle the special issues that attend the analysis of biological time series.

### Philosophical background

Defining, discovering and exploiting causal relations have a long and enduring history (Bunge, 2009). Examples of current philosophical debates about causality can be found in Woodward (Woodward, 2003) and Cartwright (2007). An important concept, stressed by Woodward, is that a cause is something that “makes things happen”. Cartwright, on the other hand (Cartwright, 2007), argues for the need to separate the definition, discovery and use of causes; stresses the pluralism of the concept of cause and argues for the use of “thick causal concepts”. An example of what she calls a “thin causal claim” would be that “activity in the retina causes activity in V1”—represented as a directed arrow from one structure to the other. Instead, it might be more useful to say that the Retina is mapped via a complex logarithmic transform to V1 (Schwartz, 1977). A “thick causal” explanation tries to explain how information is actually transmitted. For a different perspective see Glymour (2009). It may be that both thin and thick causal concepts are useful when characterizing complex systems.

Despite philosophical disagreements about the study of causality, there seems to be a consensus that causal modeling is a legitimate statistical enterprise (Cox and Wermuth, 2004, Frosini, 2006, Pearl, 2003). One can clearly differentiate two current streams of statistical causal modeling; one based on Bayesian dependency graphs or graphical models which has been labeled as “Structural Causal Modeling” by White and Lu (2010). The other, apparently unrelated, approach rests on some variant of Granger Causality for which we prefer the terms WAGS influence^8^ for reasons stated below. WAGS influence modeling appeals to an improved predictability of one time series by another. We will describe these two streams of modeling, which leads us to anticipate their combination in a third line of work, called Dynamic Structural Systems (White and Lu, 2010).

### Structural causal modeling: graphical models and Bayes–Nets

Structural Causal Modeling originated with Structural Equation Modeling (SEM) (Wright, 1921) and is characterized by the use of graphical models, in which direct causal links are encoded by directed edges in the graph (Lauritzen, 1996, Pearl, 2000, Spirtes et al., 2000). Ideally these edges can be given a mechanistic interpretation (Machamer et al., 2000). Using these graphs, statistical procedures then discover the best model (graph) given the data (Pearl, 2000, Pearl, 2003, Spirtes et al., 2000). As explained in the previous section, the “best” model has the highest evidence. There may be many models with the same evidence; in this case, the statistical search produces an equivalence class of models with the same explanatory power. With regard to effective connectivity, the multiplicity of possibly equivalent models has been highlighted by Ramsey et al. (2010).

This line of work has furnished Statistical Causal Modeling with a rigorous foundation and specific graphical procedures such as the “Back-door” and “Front-door” criteria, to decide whether a given causal model explains observational data. Here, causal architectures are encoded by the structure of the graph. In fMRI studies these methods have been applied by Ramsey et al. (2010) to estimate directionality in several steps, first looking for “unshielded colliders” (paths of the form A → B ← C) and then finding out what further dependencies are implied by these colliders. We now summarize Structural Causal Modeling, as presented by Pearl (2000).

One of the key concepts in Pearl's causal calculus is *interventional probabilities*, which he denotes *p*(*x*_\ *i*_|*do*(*X*_*i*_ = *x*_*i*_)) or more simply *p*(*x*_\ *i*_|*do*(*x*_*i*_)), which are distinct from conditional probabilities *p*(*x*_\ *i*_|*X*_*i*_ = *x*_*i*_). Pearl highlights the difference between the *action do*(*X*_*i*_ = *x*_*i*_) and the *observation X*_*i*_ = *x*_*i*_. Note that observing *X*_*i*_ = *x*_*i*_ provides information both about the children and parents of *X*_*i*_ in a directed acyclic graph (DAG^9^). However, whatever relationship existed between *X*_*i*_ and its parents prior to action, this relationship is no longer in effect when we perform the action *do*(*X*_*i*_ = *x*_*i*_). *X*_*i*_ is held fixed by the action *do*(*X*_*i*_ = *x*_*i*_), and therefore cannot be influenced. Thus, inferences based on evaluating *do*(*X*_*i*_ = *x*_*i*_) are different in nature from the usual conditional inference. Interventional probabilities are calculated via a *truncated factorization*; i.e. by conditioning on a “mutilated graph”, with the edges (links) from the parents of *X*_*i*_ removed:

(7) $$p\left(x_{\backslash i}|do(x_{i})\right)=\prod_{j\neq i}p\left(x_{j}|pa_{j}\right)=\frac{p\left(x\right)}{p\left(x_{i}|pa_{i}\right)}\text{.}$$

Here, *pa*_*j*_ denotes the set of all the parents of the *j*th node in the graph and *p*(*x*) is the full joint distribution. Such interventional probabilities exhibit two properties:

(8) $$\left\{ \begin{array}{l} P1:p\left(x_{i}|do\left(pa_{i}\right)\right)=p\left(x_{i}|pa_{i}\right)\\ P2:p\left(x_{i}|do\left(pa_{i}\right),do\left(s\right)\right)=p\left(x_{i}|do\left(pa_{i}\right)\right) \end{array}\right.$$

for all *i* and for every subset *S* of variables disjoint of {*X*_*i*_, *PA*_*i*_}. Property 1 renders every parent set *PA*_*i*_ exogenous relative to its child *X*_*i*_, ensuring that the conditional *p*(*x*_*i*_|*pa*_*i*_) probability coincides with the effect (on *X*_*i*_) of setting *PA*_*i*_ to *pa*_*i*_ by external control. Property 2 expresses the notion of invariance: once we control its direct causes *PA*_*i*_, no other interventions will affect the probability of *X*_*i*_. These properties allow us to evaluate the (probabilistic) effect of interventions from the definition of the joint density *p*(*x*) associated with the pre-intervention graph.

This treatment of interventions provides a semantics for notions such as “causal effects” or “causal influence”. For example, to see whether a variable *X*_*i*_ has a causal influence on *X*_*j*_, we compute (using the truncated factorization in Eq. (7)) the marginal distribution of *X*_*j*_ under the actions *do*(*X*_*i*_ = *x*_*i*_) and check whether that distribution is sensitive to *x*_*i*_. It is easy to see that only descendants of *X*_*i*_ can be influenced by *X*_*i*_; deleting the factor *p*(*x*_*i*_|*pa*_*i*_) from the joint distribution turns *X*_*i*_ into a root node^10^ in the mutilated graph. This can be contrasted with (undirected) probabilistic dependencies that can be deduced from the factorization of the joint distribution *per se*. These dependencies can be thought as (non-causal and non-directed) correlations among measured variables that can be predicted on the basis of the structure of the network.

**Fig. 5:**
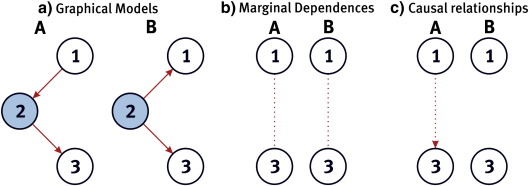
The missing region problem. a) Two typical graphical models including a hidden node (node 2).b) Marginal dependence relationships implied by the causal structure depicted in (a), after marginalizing over the hidden node 2; the same moral graph can be derived from directed (causal) graphs A and B. c) Causal relationships implied by the causal structure depicted in (a), after marginalizing over the hidden node 2. Note that these are perfectly consistent with the moral graph in (b), depicting (non causal) statistical dependencies between nodes 1 and 3, which are the same for both A and B.

In the context of brain connectivity, the measures of interventional and conditional probabilities map onto the notions of effective connectivity and functional connectivity respectively. Let us consider two typical situations that arise in the context of the missing region problem. These are summarized in Fig. 5.

Consider Fig. 5a. In situation A, node 1 influences node 2, which influences node 3. That is, the causal effect of 1 on 3 is *mediated by 2*. The joint distribution of the graphical causal model can be factorized as *p*_*A*_(*x*) = *p*(*x*_3_|*x*_2_)*p*(*x*_2_|*x*_1_)*p*(*x*_1_). In situation B, both 1 and 3 have a common cause: node 2 influences both 1 and 3. The joint distribution of this graphical causal model can then be factorized as: *p*_*B*_(*x*) = *p*(*x*_1_|*x*_2_)*p*(*x*_3_|*x*_2_)*p*(*x*_2_). It is easy to prove that in both cases (A and B), 1 and 3 are *conditionally independent* given 2; i.e., *p*(*x*_1_, *x*_3_|*x*_2_) = *p*(*x*_1_|*x*_2_)*p*(*x*_3_|*x*_2_). This means that observing node 1 (respectively 3) does not convey additional information about 3 (respectively 1), once we know 2. Furthermore, note that 1 and 3 are actually *marginally dependent*; i.e., $p\left(x_{1},x_{3}\right)=\int p\left(x\right)dx_{2}\neq p\left(x_{1}\right)p\left(x_{3}\right)$. This means that whatever value *X*_2_ might take, *X*_1_ and *X*_3_ will be correlated. Deriving the marginal independencies from the DAG produces an undirected graph (see, e.g., Fig. 5b). This undirected graph is called a *moral graph* and its derivation is called the *moralization* of the DAG. For example, moralizing the DAG A produces a fully connected moral graph.

In brief, both situations (A and B) are similar in terms of their statistical dependencies. In both situations, functional connectivity methods would recover the conditional independence of nodes 1 and 3 if node 2 was observed, and their marginal dependence if it is not (see Fig. 5b).However, the situations in A and B are actually very different in terms of the causal relations between 1 and 3. This can be seen using the interventional probabilities defined above: let us derive the interventional probabilities expressing the causal influence of node 1 onto node 3 (and reciprocally) in situation A:

(9) $$\begin{array}{c} p_{A}\left(x_{3}|do\left({\tilde{x}}_{1}\right)\right)=\int p_{A}\left(x_{2},x_{3}|do\left({\tilde{x}}_{1}\right)\right)dx_{2}\\ =\int p\left(x_{3}|x_{2}\right)p\left(x_{2}|{\tilde{x}}_{1}\right)dx_{2}\\ =p\left(x_{3}|{\tilde{x}}_{1}\right) \end{array}$$

(10) $$\begin{array}{c} p_{A}\left(x_{1}|do\left({\tilde{x}}_{3}\right)\right)=\int p_{A}\left(x_{1},x_{2}|do\left({\tilde{x}}_{3}\right)\right)dx_{2}\\ =p\left(x_{1}\right)\int p\left(x_{2}|x_{1}\right)dx_{2}\\ =p\left(x_{1}\right)\text{.} \end{array}$$

Eq. (8) simply says that the likelihood of any value that *x*_3_ might take is dependent upon the value ${\tilde{x}}_{1}$ that we have fixed for *x*_1_ (by intervention). In contradistinction, Eq. (9) says that the likelihood of any value that *x*_1_ might take is independent of *x*_3_. This means that node 1 has a causal influence on node 3, i.e. there is a directed (mediated through 2) causal link from 1 to 3. The situation is quite different in B:

(11) $$\begin{array}{c} p_{B}\left(x_{3}|do\left({\tilde{x}}_{1}\right)\right)=\int p_{B}\left(x_{2},x_{3}|do\left({\tilde{x}}_{1}\right)\right)dx_{2}\\ =\int p\left(x_{3}|x_{2}\right)p\left(x_{2}\right)dx_{2}\\ =p\left(x_{3}\right)\\ p_{B}\left(x_{1}|do\left({\tilde{x}}_{3}\right)\right)=\int p_{B}\left(x_{1},x_{2}|do\left({\tilde{x}}_{3}\right)\right)dx_{2}\\ =\int p\left(x_{1}|x_{2}\right)p\left(x_{2}\right)dx_{2}\\ =p\left(x_{1}\right)\text{.} \end{array}$$

This shows that nodes 1 and 3 are not influenced by intervention on the other. This means that here, there is no causal link between 1 and 3.This is summarized in Fig. 5c, which depicts the corresponding ‘effective’ causal graphs, having marginalized over node 2.

Causal calculus provides a simple but principled perspective on the “missing region” problem. It shows that effective connectivity analysis can, in certain cases, address a subset of brain regions (subgraph), leaving aside potential variables (e.g., brain regions) that might influence the system of interest. The example above makes the precise confines of this statement clear: one must be able to perform interventional actions on source and target variables. Given that the principal ‘value-setting’ interventions available to us in cognitive neuroscience are experimental stimulus manipulations, our capacity for such interventions are generally limited to the primary sensory cortices. Intervention beyond sensorimotor cortex is much more difficult; although one could employ techniques such as transcranial magnetic stimulation (TMS) to perturb activity in superficial cortical areas. However, the perturbation in TMS is unnatural and known to induce compensatory changes throughout the brain rather than well-defined effects in down-stream areas.

The same undirected graph can be derived from the moralization of a set of DAGs (c.f. from Figs. 5a and b). This set contains a (potentially infinite) number of elements, and is referred to as the *equivalent class*. As stated by Pearl, the identification of causal (i.e., interventional) probabilities from observational data requires additional assumptions or constraints (see also Ramsey et al., 2010). Pearl mentions two such critical assumptions: (i) *minimality* and (ii) *structural stability*. Minimality appeals to complexity minimization, when maximizing model evidence (c.f., Occam's razor). In brief, among a set of causal models that would explain the observed data, one must choose the simplest (e.g., the one with the fewest parameters). Structural stability (also coined ‘faithfulness’) is a related requirement that is motivated from the fact that an absence of causal relationships is inferred from an observed absence of correlation. Therefore, if no association is observed, it is unlikely to be due to the particular instantiation of a given model for which this independence would be predicted (see below). Rather, it is more likely to be explained in terms of a model that would predict, for any parameter setting, the observed absence of correlation. This clearly speaks to the convergent application, mentioned above, of data driven exploratory approaches that scan the largest model space possible for correlations to be explained and a model driven confirmatory approach that appeal to structural stability: Within a Bayesian setting, we usually specify a prior distribution *p*(*θ*|*m*) over model parameters, which are usually assumed to be independent. This is justified when the parameters represent mechanisms that are free to change independently of one another—that is, when the system is structurally stable. In other terms, the use of such prior favors structurally stable models. In most cases, stability and minimality are sufficient conditions for solving the structure discovery inverse problem in the context of observational data. If this is not sufficient to reduce the cardinality of the equivalent class, one has to resort to experimental interventions.^11^ Within the context of Neuroimaging, this would involve controlling the system by optimizing the experimental design in terms of the psychophysical properties of the stimuli and/or through direct biophysical stimulation (e.g., transcranial magnetic stimulation – TMS – or deep brain stimulation—DBS).

### Summary

The causal calculus based on graphical models has some important connections to the distinction between functional and effective connectivity and provides an elegant framework in which one can deal with interventions. However, it is limited in two respects. First, it is restricted to discovering conditional independencies in *directed acyclic graphs*. This is a problem because the brain is a directed *cyclic* graph—every brain region is reciprocally connected (at least polysynaptically) and every computational theory of brain function rests on some form of reciprocal or reentrant message passing. Second, the calculus ignores time: Pearl argues that what he calls a ‘causal model’ should rest upon *functional relationships* between variables, an example of which is structural equation modeling (SEM). However, these functional relationships cannot deal with (cyclic) feedback loops. In fact, DCM was invented to address these limitations, after evaluating structural causal modeling for fMRI time-series. This is why it was called *dynamic* causal modeling to distinguish it from *structural* causal modeling (Friston et al., 2003). Indeed, Pearl (2000) argues in favor of dynamic causal models, when attempting to identify what physicists call hysteresis effects, whereby the causal influence depends upon the history of the system. Interestingly, the DAG limitation can be finessed by considering dynamics and temporal precedence within structural causal modeling. This is because the arrow of time turns directed cyclic graphs into directed acyclic graphs, when the nodes are deployed over successive time points. This leads us to an examination of prediction-based measures of functional relations.

### WAGS influence

The second stream of statistical causal modeling is based on the premise that a cause must precede and increase the predictability of its consequence. This type of reasoning can be traced back at least to Hume (Triacca, 2007) and is particularly popular in time series analysis. Formally, it was originally proposed (in an abstract form) by Wiener (1956) (see Appendix A) and introduced into data analysis by Granger (1963). Granger emphasized that increased predictability is a necessary but not sufficient condition for a causal relation to exist. In fact, Granger distinguished between true causal relations and “prima facie” causal relations (Granger, 1988); the former only to be inferred in the presence of “knowledge of the state of the whole universe”. When discussing “prima facie causes” we recommend the use of the neutral term “influence” in agreement with other authors (Commenges and Gégout-Petit, 2009, Commenges and Gégout-Petit, 2009). Additionally, it should be pointed out that around the same time as Grangers work, Akaike, 1968, Schweder, 1970 introduced similar concepts of influence, prompting us to refer to “WAGS influence modeling” (for **W**iener–**A**kaike–**G**ranger–**S**chweder). This is a generalization of a proposal by Aalen (1987) and Aalen and Frigessi (2007) who were among the first to point out the connections between the Granger and Shweder concepts.

An unfortunate misconception in Neuroimaging identifies WAGS influence modeling (WAGS for short) with just one of the specific proposals (among others) dealt with by Granger; namely, the discrete-time linear Vector Autoregressive Model (VAR). This simple model has proven to be a useful tool in many fields, including Neuroimaging—the latter work well documented in Bressler and Seth (2010). However, this restricted viewpoint overlooks the fact that WAGS has dealt with a much broader class of systems:

1. Classical textbooks, such as Lutkephol (2005), show how WAGS can applied VAR models, infinite order VAR, impulse response functions, Vector Autoregressive Moving Average models (VARMA), *etc*.
2. There are a number of nonlinear WAGS methods that have been proposed for analyzing directed effective connectivity (Freiwald et al., 1999, Solo, 2008, Gourieroux et al., 1987, Marinazzo et al., 2010, Kalitzin et al., 2007)
3. Early in the econometrics literature, causal modeling was extended to linear and nonlinear random differential equations in continuous time (Bergstrom, 1988). These initial efforts have been successively generalized (Aalen, 1987, Commenges and Gégout-Petit, 2009, Comte and Renault, 1996, Florens and Fougere, 1996, Gill and Petrović, 1987, Gégout-Petit and Commenges, 2010, Mykland, 1986, Petrović and Stanojević, 2010) to more inclusive types of dynamical systems.
4. Schweder (1970) describes WAGS concepts for counting processes in continuous, time which has enjoyed applications in Survival Analysis—a formalism that could well be used to model interactions expressed in neural spike train data.

We now give an intuitive explanation of some of these definitions (the interested reader can refer to the technical literature for more rigorous treatments). Let us again consider triples of (possibly vector) time series *X*_1_(*t*), *X*_2_(*t*), *X*_3_(*t*), where we want to know if time series *X*_1_(*t*) is influenced by time series *X*_2_(*t*) conditional on *X*_3_(*t*). This last variable can be considered as any time series to be controlled for (if we were omniscient, the “entire universe”!). Let *X*[*a*, *b*] = {*X*(*t*)|*t* ∈ [*a*, *b*]} denote the history of a time series in the discrete or continuous time interval [*a*, *b*]. There are several types of influence. One distinction is based on what part of the present or future of *X*_1_(*t*) can be predicted by the past or present of *X*_2_(*τ*) *τ* < *t*. This leads to the following classification:

• If *X*_2_(*τ*): *τ* < *t*, can influence any future value of *X*_1_(*s*) for s > t, then it is a global influence.
• If *X*_2_(*τ*) *τ* < *t*, can influence *X*_1_(*t*) it is a local influence.
• If *X*_2_(*τ*) *τ* = *t* can influence *X*_1_(*t*) it is a contemporaneous influence.

**Fig. 6:**
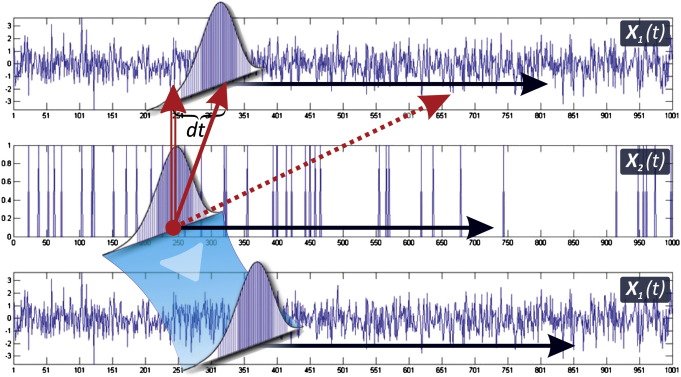
Wiener–Akaike–Granger–Schweder (WAGS) Influences. This figure illustrates the different types of WAGS influence measures. In the middle *X*_2_(*t*) a continuous time point process, which may be influencing the differentiable continuous time process *X*_1_(*t*) (top and bottom) This process may have local influence (full arrows), which indicate predictability in the immediate future (d*t*), or global influence (dashed arrow) at any set of future times. If predictability pertains to the whole probability distribution, this is a strong influence (bottom), and a weak influence (top) if predictability is limited to the moments (e.g., expectation) of this distribution.

Another distinction is whether one predicts the whole probability distribution (**strong** influence) or only given moments (**weak** influence). These two classifications give rise to six types of influence as schematized in Fig. 6 and Table 4, Table 5. Briefly, the formal definitions are as follows.

**Table 4:** Conditional Independence relations.

	Strong (Probability Distribution)	Weak (Expectation)
Global (for all horizons)	Strongly, Conditionally, Globally, independence (not SCGi)	Weakly, Conditionally, Globally, independence (not WCGi)
Local (Immediate future)	Strongly, Conditionally, Locally, independence (not SCLi)	Weakly, Conditionally, Globally, independence (not WCLi)
Contemporaneous	Strongly, Conditionally, Contemporaneously, independence (not SCCi)	Weakly, Conditionally, Contemporaneously, independence (not WCCi)

**Table 5:** Types of Influence defined by absence of the corresponding independences in Table 4.

	Strong (Probability Distribution)	Weak (Expectation)
Global ( for all horizons)	Strongly, Conditionally, Globally, influence (SCGi)- Strong Granger or Sims influence	Weakly, Conditionally, Globally, influence (WCGi)- Weak Granger or Sims influence
Local (Immediate future)	Strongly, Conditionally, Locally, influence (SCLi) - Influence (Possibly indirect)	Weakly, Conditionally, Globally, influence (WCLi) - Direct Influence
Contemporaneous	Strongly, Conditionally, Contemporaneously, influence (SCCi)	Weakly, Conditionally, Contemporaneously, influence (WCCi)

*X*_1_(*t*) is strongly, conditionally, and globally independent of *X*_2_(*t*) given *X*_3_(*t*) (not SCGi), if

(12) $$P\left(X_{1}(\infty ,t]|X_{1}(t,-\infty ],X_{2}(t,-\infty ],X_{3}(t,-\infty ]\right)=P\left(X_{1}(\infty ,t]|X_{1}(t,-\infty ],X_{3}(t,-\infty ]\right)\text{.}$$

When this condition does not hold we say *X*_2_(*t*) strongly, conditionally, and globally influences (SCGi) *X*_1_(*t*) given *X*_3_(*t*). Note that the whole future of *X*_*t*_ is included (hence the term “global”). And the whole past of all time series is considered. This means these definitions accommodate non-Markovian processes (for Markovian processes, we only consider the previous time point). Furthermore, these definitions do not depend on an assumption of linearity or any given functional form (and are therefore applicable to any of the state equations in Table 2). Note also that this definition is appropriate for point processes, discrete and continuous time series, even for categorical (qualitative valued) time series. The only problem with this formulation is that it calls on the whole probability distribution and therefore its practical assessment requires the use of measures such as mutual information.

*X*_1_(*t*) is weakly, conditionally and globally independent of *X*_2_(*t*) given *X*_3_(*t*) (not WCGi), if

(13) $$E\left[X_{1}(\infty ,t]|X_{1}(\infty ,t],X_{2}(t,-\infty ],X_{3}(t,-\infty ]\right]=E\left[X_{1}(\infty ,t]|X_{1}(t,-\infty ],X_{3}\left(t,-\infty \right]\right]\text{.}$$

If this condition does not hold we say *X*_2_(*t*) weakly, conditionally and globally influences (WCGi) *X*_1_(*t*) given *X*_3_(*t*). This concept extends to any number of moments (such as the variance of the process). There are a number of relations between these concepts: not SCGi implies not WCGi for all its moments and the converse is true for influences (WCGi implies SCGi), but we shall not go into details here; see Florens and Mouchart, 1985, Florens, 2003, Florens and Fougere, 1996, and Florens and Mouchart (1982).

Global influence refers to influence at any time in the future. If we want to capture the idea of immediate influence we use the local concepts defined above. The concepts of strong and weak local influence have very simple interpretations if we are modeling in discrete time and events occur every Δt. To see this, consider the expectation based weak conditionally local independence (not WCLi) in discrete time:

(14) $$E\left[X_{1}\left(t+\Delta t\right)|X_{1}[t,-\infty ],X_{2}[t,-\infty ],X_{3}[t,-\infty ]\right]=E\left[X_{1}\left(t+\Delta t\right)|X_{1}[t,-\infty ],X_{3}[t,-\infty ]\right]\text{.}$$

If this condition does not hold we have that *X*_2_(*t*) weakly, conditionally and locally influences (WCLi) *X*_1_(*t*) given *X*_3_(*t*). Strong local concepts are defined similarly by considering conditional independences. For the usual discrete time, real valued time series of Neuroimaging, all these concepts are equivalent as shown by Florens and Mouchart (1982) and Solo (2007). As an example, consider the multivariate autoregressive model of the previous section

(15) $$X\left(t+\Delta t\right)=\sum_{k=1}^{p}A_{k}X\left(t-(k-1)\Delta t\right)+e\left(t+\Delta t\right)$$

with the innovation term *e*_*t* + *Δt*_ being GWN with covariance matrix *Σ* : = *Σ*_*discrete*_. For this familiar case $E\left[X[t+\Delta t]|X[t,-\infty ]\right]=\sum_{k}A_{k}X\left(t-(k-1)\Delta t\right)$, and analyzing influence reduces to finding which coefficients of the autoregressive coefficients are zero. However, in continuous time there is a problem when Δt→0, since the stochastic processes we are dealing with are at least almost surely continuous and $\lim_{\Delta t\to 0}E\left[X_{1}\left(t+\Delta t\right)|X_{1}\left[t,-\infty \right],X_{2}\left[t,-\infty \right],X_{3}\left[t,-\infty \right]\right]=\lim_{\Delta t\to 0}E\left[X_{1}(t+\Delta |)\right]$ is trivially satisfied (limits are now taken in the sense of a quadratic mean) because the *X*_1_(*t*) process is path continuous—it will only depend on itself. To accommodate this situation instead we shall use the following definition for not WCLi (Commenges and Gégout-Petit, 2009, Comte and Renault, 1996, Florens and Fougere, 1996, Renault et al., 1998):

(16) $$\begin{array}{c} \underset{\Delta t\to 0}{\text{lim}E}\left[\frac{X_{1}\left(t+\Delta t\right)-X_{1}\left(t\right)}{\Delta t}|X_{1}(t,-\infty ],X_{2}(t,-\infty ],X_{3}(t,-\infty ]\right]\\ =\underset{\Delta t\to 0}{\text{lim}E}\left[\frac{X_{1}\left(t+\Delta t\right)-X_{1}\left(t+\Delta t\right)}{\Delta t}|X_{1}(t,-\infty ],X_{3}(t,-\infty ]\right]\text{.} \end{array}$$

As noted by Renault et al. (1998) (whom we follow closely here), for finite Δt this is equivalent to the usual definitions. Now how does this definition relate to the linear SDE in Eq. (3)?

For three time series:

(17) $$\left(\begin{array}{l} dX_{1}\left(t\right)\\ d{\text{X}}_{2}\left(t\right)\\ d{\text{X}}_{3}\left(t\right) \end{array}\right)=A\left[\begin{array}{l} X_{1}\left(t\right)\\ X_{2}\left(t\right)\\ X_{3}\left(t\right) \end{array}\right]dt+dB\left(t\right)\text{.}$$

Integrating from *t* to Δt, we have

$$\begin{array}{l} X_{1}\left(t+\Delta t\right)-X_{1}\left(t+\Delta t\right)=\int_{t}^{t+\Delta t}\left[a\left(1,1\right)X\left(\tau \right)+a\left(1,2\right)X_{2}\left(\tau \right)+a\left(1,3\right)X_{3}\left(\tau \right)+\right]d\tau +\sigma_{\mathrm{bb}}\left(B_{1}\left(t+\Delta t\right),-B_{2}\left(t\right)\right)\\ \Rightarrow \\ \underset{\Delta t\to 0}{\text{lim}E}\left[\frac{X_{1}\left(t+\Delta t\right)-X_{2}\left(t\right)}{\Delta t}|X_{1}\left(t\right),X_{2}\left(t\right),X_{3}\left(t\right)\right]=a\left(1,1\right)X_{2}\left(\tau \right)+a\left(1,2\right)X_{2}\left(\tau \right)+a\left(1,3\right)X_{3}\left(\tau \right)\text{.} \end{array}$$

This shows that, in effect, the detection of an influence will depend on whether the coefficients of the matrix *A* are zero or not. For nonlinear systems this holds with the local linear approximation. This treatment highlights the goal of WAGS, like structural causal modeling, is to detect conditional independencies; in this (AR) example, weak and local.

The issue of contemporaneous influence measures is quite problematic. In discrete time, it is clear that the covariance matrix of two or more time series may have cross-covariances that are due to an “environmental” or missing variable *Z*(*t*). This was discussed by Akaike and a nice example of this effect is described in Wong and Ozaki (2007), which also explains the relation of the Akaike measures of influence to others used in the literature. For continuous time (Comte and Renault, 1996) define strong (second order) conditional contemporaneous independence (not SCCi) if:

(18) $$\mathrm{cov}\left[X_{1}\left(\infty ,t\right],X_{2}\left(\infty ,t\right]|X_{1}\left[t,-\infty \right)X_{2}\left[t,-\infty \right),X_{3}\left[t,-\infty \right)\right]=0.$$

Note that this is the same definition for continuous time as for the discrete AR example (Eq. (15)) and is equivalent to requiring that the elements of the corresponding innovation covariance matrix *Σ* be zero. These authors then went on to define weak contemporaneous conditional independence (not WCCi) if:

(19) $$\underset{\Delta t\to 0}{\text{lim}}\left\{\text{cov},\left[X_{1}\left(t+\Delta t\right),X_{2}\left(t+\Delta t\right)|X_{1}\left[t,-\infty \right),X_{2}\left[t,-\infty \right),X_{3}\left[t,-\infty \right)\right]\right\}=0.$$

In the absence of these conditions we have strong (weak) contemporaneous conditional influences which are clearly non-directional. In his initial paper (Granger, 1963) defined a contemporaneous version of his influence measure in discrete time. Much later, (Geweke, 1984)decomposed his own WAGS measure into a sum of parts, some depending on lag information and others reflecting contemporaneous (undirected) influences, see in these C&C (Bressler and Seth, 2010). However, Granger (in later discussions) felt that if the system included all relevant time series this concept would not be valid, unless these influences were assigned a directionality (see Granger, 1988, pp. 204–208). In this sense, he was proposing a Structural Equation Modeling approach to the covariance structure of the autoregressive model innovations. As will be mentioned below (WAGS influence section) this is something that has been explored in the econometrics literature by Demiralp and Hoover, 2008, Moneta and Spirtes, 2006, but not to our knowledge in Neuroimaging.

#### More general models

As we have seen, strong global measures of independence are equivalent to conditional independence and are therefore applicable to very general stochastic processes. For weak local conditional independence, the situation is a little more difficult and we have given examples, which involve a limit in the mean of a derivative-type operator expression. The more general theory, too technical to include here, entails successive generalizations by Mykland, 1986, Aalen, 1987, Commenges and Gégout-Petit, 2009, and Gégout-Petit and Commenges (2010). The basic concept can be stated briefly as follows (we drop conditioning on a third time series for convenience). Suppose we have stochastic processes that are semi-martingales of the form, *X*(*t*) = *P*^*X*^(*t*) + *M*^*X*^(*t*). Here *P*^*X*^(*t*) is a predictable stochastic process^12^ of bounded variation, which is known as the “compensator” of the semi-martingale, and *M*_*t*_^*X*^ is a martingale.^13^ Predictability is the key property that generalizes Wiener's intuition. The martingale component is the unpredictable part of the stochastic process we are interested in.^14^ Now suppose we have two stochastic processes *X*(*t*) and *W*(*t*). If:

1. The martingales *M*^*X*_1_^ and *M*^*X*_2_^ are orthogonal (no contemporaneous interactions).
2. *P*^*X*_1_^(*t*) is measurable^15^ with respect to *X*_1_[*t*, − *∞*] only (without considering *X*_2_(*t*)).

then *X*_1_(*t*) is said to be weakly locally independent of *X*_2_(*t*). In Gégout-Petit and Commenges (2010) the concept of ~ WLCi is generalized to a general class of random phenomena that include random measures, marked point processes, diffusions, and diffusions with jumps, covering many of the models in Table 2. In fact, this theory may allow unification of the analysis of random behavioral events, LFP, spike recordings, and EEG, just to give a few examples.

#### Direct influence

**Fig. 7:**
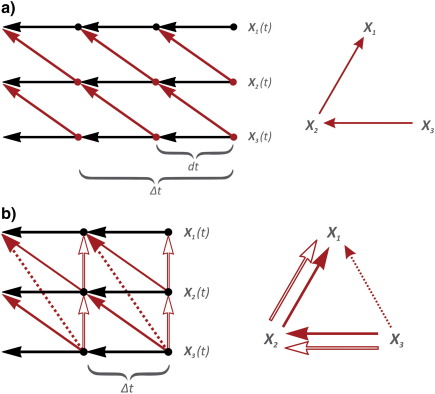
The missing time problem. This figure provides a schematic representation of spurious causality produced by sub-sampling. a) Three time series *X*_1_(*t*), *X*_2_(*t*), and *X*_3_(*t*) are shown changing at an “infinitesimal” time scale with steps d*t*, as well as at a coarser sampled time scale with set ∆*t*. Each time series, influences itself at later moments. In the example *X*_3_(*t*) directly influences *X*_2_(*t*), with no direct influence on *X*_1_(*t*). In turn *X*_2_(*t*) directly influences *X*_1_(*t*), with no direct influence on *X*_3_(*t*). Finally *X*_1_(*t*) does not influence either *X*_3_(*t*) nor *X*_2_(*t*). There are no contemporaneous influences. b) When only observing at the coarser time scale ∆*t*, spurious contemporaneous influences (mediated by intermediate nodes) appear between *X*_2_(*t*) and *X*_1_(*t*) and between *X*_3_(*t*) and *X*_2_(*t*). In addition a spurious direct influence appears between *X*_3_(*t*) and *X*_1_(*t*). The graphical representations of the true and spurious causal relations are to the right of each figure where an arrow represents direct influence and a double arrow represents contemporaneous influence. Estimating these spurious influences can only be avoided by explicitly modeling their effect from continuous models or using models such as VARMA models which are resistant to this phenomena.

Weak local independence might be considered an unnecessarily technical condition for declaring the absence of an influence; in that strong (local or global) influence measures should be sufficient. An early counterexample of this was provided by Renault et al. (1998), where they considered a model where *X*(*t*) is ~ WCLi of *W*(*t*), given *Z*(*t*). See Fig. 7 for an illustration of this divergence between local and global influences. This has lead Commenges and Gégout-Petit (2009) to define WCLi as the central concept for “direct influence” whereas SCGi is an influence that can be mediated directly or indirectly through other time series.

An important point here is the degree to which the definition of WAGS influence depends on the martingale concept or, indeed, on that of a stochastic process. As discussed in The observation equation section, there are a number of instances in which Markovian models developed for financial time series may not apply for Neuroimaging data. However, the concepts are probably generally valid, as we shall illustrate with some examples:

• The analytical random processes used in generalized coordinates are quite different from those usually studied in classical SDE theory but have been known for a long time (Belyaev, 1959). In fact, there has been quite a lot of work on their predictability (Lyman et al., 2000) and indeed there is even work on VARMA modeling of this type of process (Pollock, 2010).
• We have already seen that the definitions of influence do not depend on Markovian assumptions as noted by Aalen (1987).
• The use of deterministic bilinear systems in DCM (Penny et al., 2005) suggests that (non-stochastic) ODEs may be incorporated into the WAGS framework. This sort of assimilation has in fact been proposed by Commenges and Gégout-Petit (2009) as a limiting case of the definition based on semi-martingales above. Extensions of the definition might be required when dealing with chaotic dynamics but, even here, measure theoretic definitions are probably valid.^16^ An interesting discussion of determinism versus stochastics can be found in Ozaki (1990).

The use or development of WAGS theory for systems that were not initially considered by the aforementioned papers may well be a fruitful area of mathematical research. In particular, WAGS may be especially powerful when applied to processes defined on continuous spatial manifolds (Valdes-Sosa, 2004, Valdés-Sosa et al., 2006).To our knowledge, WAGS has yet to be developed for the case of continuous time and space models; for example, those expressed as stochastic or random Partial Differential Equations.

#### Testing and measuring WAGS influence

Above, we have covered different types of WAGS influence. With these definitions in place we now distinguish between testing for the presence of an influence (inference on models) and estimating the strength of the influence (inference on parameters). There is an extensive literature on this, which we shall not go into here. Examples of testing versus measuring for discrete time VAR models include the Dickey–Fuller test and the Geweke measure of influence. In the electrophysiological literature, there are a number of measures proposed. A review and a toolbox for these measures can be found in Seth (2009). From the point of view of effective connectivity, many of these measures have an uncertain status. This is because effective connectivity is only defined in relation to a generative model. In turn, this means there are only two quantities of interest (that permit inference on models and parameters respectively): the relative evidence for a model with and without a connection and the estimate (conditional density over) the connection parameter. For DCM the first quantity is the Bayes factor and for GCM it is the equivalent likelihood ratio (Granger causal F-statistics). In DCM, the conditional expectation of the parameter (effective connectivity) measures the strength, while for GCM this is the conditional estimate of the corresponding autoregression coefficient. Other measures (e.g., partial directed coherence) are simply different ways of reporting these conditional estimates. The next section explores the use of WAGS measures of direct and indirect effects within the Structural Causal modeling framework, thus bringing together the two major strands of statistical causal modeling.

### Dynamic structural causal modeling

There have been recent theoretical efforts to embed WAGS into Structural Causal Modeling, which one could conceive of (in the language of Granger) as providing a means to find out which “prima facie causes” are actual “causes”. One of the first people to use the methods from Structural Causal Modeling was Granger himself: Swanson and Granger (1997) used Bayes-Net methods described in Spirtes et al. (2000) in combination with autoregressive modeling. Similar approaches have been adopted by Demiralp and Hoover (2008) and Moneta and Spirtes (2006), which address the search for directed contemporaneous influences mentioned above.

However, we should mention three current attempts to combine Structural Causal Modeling with WAGS influence analysis. We shall follow White in calling models that can be described by both theoretical frameworks Dynamic Structural Systems:

1. Eichler has been developing graphical time series models that are based on discrete time WAGS. Recently, in work with Didelez the formalization of interventions has been introduced and equivalents for the backdoor and front-door criteria of Structural Causality have been defined. Thus, for discrete systems, this work could result in practical criteria for defining when it is possible to infer causal structure from WAGS in discrete time.
2. White has created a general formalism for Dynamical Structural Systems (White and Lu, 2010) based on the concept of settable systems (White and Chalak, 2009), which supports model optimization, equilibrium and learning. The effects of intervention are also dealt with explicitly.
3. Commenges and Gégout-Petit (2009) have also proposed a general framework for causal inference that combines elements of Bayes–Nets and WAGS influence and has been applied to epidemiology. Specifically, as mentioned above, they introduce a very general definition of WAGS that is valid for continuous/discrete time processes. This definition can be applied to a mixture of SDEs and point processes and distinguishes between direct influences and indirect influences. They then relate the definition to graphical models, with nodes connected by direct influences only and place their work in the context of General Systems Theory. Interestingly, they stress the need for an observation equation to assure causal explanatory power.

The common theme of all these efforts is to supplement predictability with additional criteria to extend WAGS influence to inference on causal mechanisms. In the words of Gégout-Petit and Commenges (2010): “A causal interpretation needs an epistemological act to link the mathematical model to a physical reality.” We will illustrate these ideas with a particular type of SSM, known as a (stochastic) dynamic causal model (DCM):

(20) $$\left\{ \begin{array}{l} \dot{x}=f\left(x,\theta ,u\right)+\omega \\ y=g\left(x,\theta \right)+\varepsilon \end{array}\right.$$

where *x* are (hidden) states of the system, *θ* are evolution parameters, *u* are the experimental control variables, *ω* are random fluctuations and *ε* is observation noise. Inverting this model involves estimating the evolution parameters *θ*, which is equivalent to characterizing the structural transition density $p\left(\dot{x}|do\left(x\right)\right)$, having accounted for observational processes.^17^ Here, time matters because it prevents instantaneous cyclic causation, but still allows for dynamics. This is because identifying the structural transition density $p\left(\dot{x}|do\left(x\right)\right)$ effectively decouples the children of *X*(*t*) (in the future) from its parents (in the past). Let us now examine a bilinear form of this model

(21) $$f\left(x\right)=Ax+\sum_{i}u_{i}B^{\left(i\right)}x+Cu+\sum_{j}x_{j}D^{\left(j\right)}x\text{.}$$

Then we have:

(22) $$\begin{array}{c} A=\lim_{x,u\to 0}\frac{\partial }{\partial x}E\left[\dot{x}|do\left(x\right)\right]\\ B^{\left(i\right)}=\frac{\partial^{2}}{\partial x\partial u_{i}}E\left[\dot{x}|do\left(x\right)\right]\\ C=\lim_{x\to 0}\frac{\partial }{\partial u_{i}}E\left[\dot{x}|do\left(x\right)\right]\\ D^{\left(j\right)}=\frac{\partial^{2}}{\partial x\partial x_{j}}E\left[\dot{x}|do\left(x\right)\right]\text{.} \end{array}$$

**Fig. 8:**
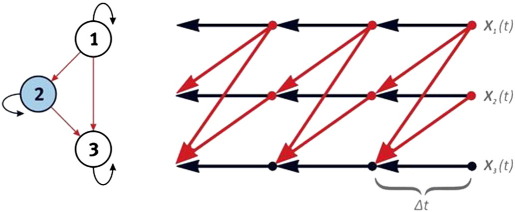
Direct and indirect effects. Causal relationships implied by the DCM given in Eq. (23). On the left the apparent graph, that includes feedback which precludes causal analysis. Note that the causal links are actually expressed through implicit delays, which makes this graph a DAG, which is seen more clearly on the right where each node is expanded at several time instants.

The meaning of *A*; i.e. the effective connectivity is the rate of change (relative to *x*) of the expected motion $E\left[\dot{X}\right]$ where *X* is held at *x* ≈ 0.^18^ It measures the *direct effect* of connections. Importantly, *indirect effects* can be derived from the effective connectivity. To make things simple, consider the following 3-region DCM depicted in Fig. 8:

(23) $$\begin{array}{l} {\dot{x}}_{1}=A_{11}x_{1}+\omega_{1}\\ {\dot{x}}_{2}=A_{21}x_{1}+A_{22}x_{2}+\omega_{2}\\ {\dot{x}}_{3}=A_{31}x_{1}+A_{32}x_{2}+A_{33}x_{3}+\omega_{3}\text{.} \end{array}$$

The effect of node 1 on node 3 is derived from the calculus of the intervention *do*(*X*_1_ = *x*_1_), where *X*_1_ is held constant at *x*_1_ but *X*_2_ is permitted to run its natural course. This intervention confirms that node 1 has both a direct and an indirect effect on node 3 (through node 2).^19^ Interestingly, indirect effects can also be derived by projecting Eq. (20) onto generalized coordinates; i.e. by deriving the evolution function of the augmented state space $\tilde{x}={\left(x,\dot{x},\ddot{x},\ldots \right)}^{T}$ (see Friston et al., 2008a,b for a variational treatment of stochastic dynamical systems in generalized coordinates). For example, deriving the left and the right hand side of the last equation in Eq. (23) with respect to time yields:

(24) $$\begin{array}{c} {\ddot{x}}_{3}={\tilde{A}}_{31}x_{1}+{\tilde{A}}_{32}x_{2}+{\tilde{A}}_{33}x_{3}+{\tilde{\omega }}_{3}\\ {\tilde{A}}_{31}=\underset{\text{direct effect}}{\underset{︸}{A_{31}\left(A_{11}+A_{33}\right)}}+\underset{\text{indirect effect}}{\underset{︸}{A_{32}A_{21}}}\\ {\tilde{A}}_{32}=A_{32}\left(A_{22}+A_{33}\right)\\ {\tilde{A}}_{33}=A_{33}A_{33} \end{array}$$

where ${\tilde{\omega }}_{3}$ lumps all stochastic inputs (and their time derivatives) together. The total effect of node 1 onto node 3 is thus simply decomposed through the above second order ODE (Eq. (24)), as the sum of direct and indirect effects. One can see that the indirect causal effect of node 1 on node 3 is proportional to the product *A*_32_*A*_21_ of the path coefficients of the links [1→2] and [2→3].This speaks to a partial equivalence of the *do* calculus and the use of generalized coordinates, when modeling both direct and mediated (indirect) effects. This is because embedding the evolution equation into a generalized coordinates of motion naturally accommodates dynamics and the respective contributions of direct/indirect connections (and correlations induced by non-Markovian state noise *ω*). However, the embedding (truncation) order has to be at least as great as the number of intermediary links to capture indirect effects.

This type of reasoning is very similar to the treatment of direct and indirect influences under WAGS influence and exemplifies a convergence of Structural Causal (Bayes-Net) Modeling and WAGS influence. One could summarize this ambition by noting the “arrow of time” converts realistic (cyclic) graphical models – that include feedback and cyclic connections – into a DAG formalism, to allow full causal inference. So what are the limits of this approach in Neuroimaging?

## Challenges for causal modeling in Neuroimaging

The papers in this C&C highlight challenges that face methods for detecting effective connectivity. These challenges arise mainly in the analysis of BOLD signals. To date, the only experimental examination of these issues is reported in the paper that originated this series (David et al., 2008). The main message from the ensuing exchanges is the need to account for the effect of the HRF; that is, to include an appropriate observation model in the analysis, along with careful evaluation of form, priors and Identifiability.

Another approach to testing the validity and limits of the methods discussed above has been through computer simulations. The results of these simulations have been mixed. A number of papers have supported the use of GCM in fMRI (Deshpande et al., 2009, Stevenson and Körding, 2010, Witt and Meyerand, 2009). Others have shown advantages for Bayes-Net methods in short time series and for GCM for longer time series (Zou et al., 2009).

An extensive set of simulations (NETSIM) has been carried out by Smith et al. (2010b) using non-stationary (Poisson-type) neural innovations in several configurations of nodes and simulating hemodynamics using the fMRI version of DCM. Many different methods were compared (apart from DCM), distinguishing between those that estimate undirected association (functional connectivity) from those that estimate “lagged” dependence (essentially a form of effective connectivity). The main conclusion was that a few undirected association methods that only used the information in the zero lag covariance matrixes perform well in identifying functional connectivity from fMRI. However, lag-based methods “perform worse”. We speculate that lag information is lost by filtering with a (regionally variable) HRF and sub-sampling. Thus one could expect that (stochastic) DCM might perform better, as supported by a comparison of SEM and DCM (Penny et al., 2004).

Interesting as these results are, several points remain unresolved. In the first place, more biophysically realistic simulations are called for, especially in the simulation of neurodynamics. The neurodynamics model in DCM for fMRI is intentionally generic, to ensure identifiability when deconvolving fMRI time-series. There is work suggesting that discrete time Vector Autoregressive Moving Average models are immune to sub-sampling and noise relative to VAR models (Amendola et al., 2010, Solo, 1986, Solo, 2007). Considering that WAGS influence modeling with VARMA models is in the standard time series textbooks (Lutkephol, 2005), it is surprising that this model has not been used in Neuroimaging, with the notable exception of (Victor Solo, 2008).

NETSIM has not yet been tested using continuous time models. The problem, as pointed out by the creators of NETSIM and (Roebroeck et al., 2005), is not only sub-sampling but the combined effect of sub-sampling and the low pass filtering of the HRF. However, these problems only pertain to AR models. Continuous time DCMs have an explicit forward model of (fast) hidden states and are not confounded by sub-sampling or the HRF, provided both are modeled properly in the DCM. The key issue is whether DCM can infer hidden states in the absence of priors (i.e., stimulus functions) that are unavailable for design-free (resting state) fMRI studies of the sort generated by NETSIM. This is an unsettled issue that will surely be followed up in the near future, with the use of biophysically more informed models and new DCM developments; e.g., DCM in generalized coordinates, stochastic DCMs and the DCM–GCM combinations that are being tested at the moment.

It should further be noted that the effect of sub-sampling (and hemodynamic convolution) are only a problem at certain spatial and temporal scales. Undoubtedly it must be a concern, when inferring the dynamics of fast neural phenomena. However, it is clear that brain activity spans many different spatial (Michael Breakspear and Stam, 2005) and temporal (Vanhatalo et al., 2005) scales. Multi-scale time series methods (including WAGS influence measures) have already been used in econometrics (Gencay et al., 2002) and could be applied in neuroscience.

One example of events that occur at a time scale that is probably sufficiently slow to allow simple (AR) WAGS influence analysis are resting state fluctuations observed in concurrent EEG/fMRI recordings. The analysis of causal relations between EEG and BOLD have been studied by several authors (Eichler, 2005, Jiao et al., 2010, Valdés-Sosa et al., 2006) and is illustrated in Fig. 4:The autoregressive coefficients of this first order sparse VAR model suggest that:

1. There are hardly any lag 0 (or contemporaneous) interactions between ROIs.
2. The only coefficients that survive the FDR threshold in the fMRI are those that link each ROI to its own past.
3. There is no influence of the fMRI on the EEG.
4. There are many, interesting interactions, among the EEG sources.
5. There are a number of influences of the EEG sources on the fMRI.

This is a consistent causal model of EEG induced fMRI modulation—valid only for the slow phenomena that survive convolution with the HRF and for the alpha band EEG activity that was investigated here. Of course there are neural phenomena that might show up at as contemporaneous at this sampling rate—but we have filtered them out. An interesting analysis of information recoverable at each scale can be found in Deneux and Faugeras (2010).

### Conclusion and suggestions for further work

1. We believe that the simulation efforts that are being carried currently out are very useful and should be extended to cover a greater realism in the neurodynamics, as well as to systematically test new proposals.
2. It will be also be important to have standardized experimental data from animals as a resource for model testing. Ideally this data set should provide intracranial recordings of possible neural drivers, BOLD-fMRI, surface EEG, diffusion MRI based structural connectivity and histological based connectivity matrices.^20^
3. There is a clear need for tools that can assess model evidence (and establish their Identifiability) when dealing with large model spaces of biophysically informed SSMs. These should be brought to bear on the issue of bounds on model complexity, imposed by the HRF convolution and sub-sampling in fMRI.
4. We foresee the following theoretical developments in Causal modeling for effective connectivity:
  a. The fusion of Bayes–Net and WAGS methods.
  b. The WAGS tools developed for combined point and continuous time stochastic processes may play an important role in the connectivity analysis of EEG/fMRI, LFP and spike train data.
  c. WAGS methods must be extended to non-standard models, among others: non-Markovian, RDE, and delay differential equations.
5. The development of exploratory (nonparametric), large scale state-space methods that are biophysically constrained and contain modality specific observation equations. This objective will depend critically on the exploration of large model spaces and is in consistent with the recent surge of methods analyzing “Ultra-High” dimensional data.
6. The explicit decomposition of multiple spatial and frequency scales.
7. Effective connectivity in the setting of Neural Field Modeling

We hope to have focused attention on these issues, within a unifying framework that integrates apparently disparate and important approaches. We are not saying that DCM and GCM are equivalent, but rather that an integration is possible within a Bayesian SSM framework and the use of model comparison methods. Our review of the field has been based on the use of state space models (SSM). While we are aware that SSMs are not the only possible framework for analyzing effective connectivity, this formulation allowed us to present a particular view that we feel will stimulate further work.

Besides reviewing current work we have discussed a number of new mathematical tools: Random Differential Equations, non-Markovian models, infinitely differentiable sample path processes, as well as the use of graphical causality models. We also considered the use of continuous-time AR and ARMA models. It may well be that some of these techniques will not live up to expectations, but we feel our field will benefit from these and other new tools that confront some of the particular challenges addressed in this discussion series.

## Appendix A Wiener's original definition of causality

This approach was first formalized by Wiener (1956) as follows.^21^ Consider a strictly stationary (possibly complex) stochastic processes^22^ *X*_1_(*t*, *ω*) defined as a collection of random variable for all integer time instants *t* and realizations *ω*. Wiener showed how to construct its “innovation”—the unit variance white noise time series *E*_1_(*t*, *ω*) which is uncorrelated with the past of *X*_1_(*t*, *ω*). The innovation *E*_2_(*t*, *ω*) can also be constructed for a second time series *X*_2_(*t*, *ω*). Now consider the random variable *K*_1_(*ω*), that part of *E*_1_(*t*, *ω*) uncorrelated with its own past and that of *E*_2_(*t*, *ω*). The variance of this random variable lies between 0 and 1 and is the degree to which the time series *X*_1_(*t*, *ω*) does not depend on the past of *X*_2_(*t*, *ω*). One minus this variance is the Wiener measure C of the causal effect of *X*_2_(*t*, *ω*) on *X*_1_(*t*, *ω*). This measure of influence was in fact expressed by Wiener as an infinite sum:

(25) $$\begin{array}{c} I_{2\to 1}^{W}=\sum_{m=1}^{\infty }{\left|\rho \left(t,t-m\right)\right|}^{2}+\sum_{m=1}^{\infty }{\left|\sum_{n=1}^{\infty }\rho \left(t,t-m\right)\bar{\rho \left(t-n,t-m\right)}\right|}^{2}+\cdots \\ \rho \left(t,s\right)=E\left[X_{1}\left(t\right),\bar{X_{2}\left(s\right)}\right] \end{array}$$

where $\bar{X\left(s\right)}$ indicates the complex conjugate of a time series.

As pointed out in Bressler and Seth (2010) this definition is not practical. We elaborate on why: First, it is limited to strictly stationary processes and involves an infinite series of moments without specification of how to perform the requisite calculations. More seriously, it only involves a finite number of series and ignores the potential confounding effect of unobserved (or latent) causes. More importantly, it adopts the “functional formulation” of von Mises that lost out to the currently predominant “stochastic formulation” of Kolmogorov and Doob (Von Mises and Doob, 1941).Nevertheless Wiener's definition has several points that deserve to be highlighted:

1. It was not limited to autoregressive models but was based on the more general Moving Average Representation (MAR).
2. Although defined explicitly for discrete time stochastic processes, the extension to continuous time was mentioned explicitly.
3. Applications in neuroscience were anticipated. In fact, Wiener elaborated on its possible use: “Or again, in the study of brain waves we may be able to obtain electroencephalograms more or less corresponding to electrical activity in different parts of the brain. Here the study of the coefficients of causality running both ways and of their analogs for sets of more than two functions f may be useful in determining what part of the brain is driving what other part of the brain *in its normal activity*”.
4. It is instructive to compare this initial definition with modern accounts of direct influence.

## Footnotes

1 Note that GCM is also used as an acronym for Granger Causal Mapping (Roebroeck et al., 2005). To avoid confusion we shall use GCM mapping or the abbreviation GCMap.

2 “Progress, far from consisting in change, depends on retentiveness. When change is absolute there remains nothing to improve and no direction is set for possible improvement: and when experience is not retained, as among savages, infancy is perpetual. Those who cannot remember the past are condemned to repeat it.” George Santayana, *The Life of Reason, Volume 1, 1905*.

3 We use the following conventions for intervals, [a,b) indicates that the left endpoint is included but not the right one and that b precedes a.

5 Who, in fact, did this not for SDE (ODE driven by Brownian noise) but for linear ODE driven by random measures, as reviewed in Bergstrom (1984).

6 Note, once again, that we use the convention $\left[t+\Delta t,t\right]$ for the time interval that goes from *t* in the past to t+Δt in the present; while not the conventional usage this will make later notation clearer.

7 Note integration should not be computed this way since it is numerically unstable, especially when the Jacobian is poorly conditioned. A list of robust and fast procedures is reviewed in Valdes-Sosa et al. (2009a).

8 It might be preferable to use a more precise term “predictability” instead of influence.

9 DAG = Directed Acyclic Graph. The word ‘graph’ refers to the mapping from the set of (factorized) joint probability densities over *X* and the actual directed acyclic graph that represents the set of conditional independencies implicit in the factorization of the joint pdf *p*(*x*).

10 A root node is a node without parents. It is marginally independent of all other variables in a DAG, except its descendents.

11 For example, the back- and front-door criteria (Pearl, 2000) can be used to optimize the intervention.

12 Roughly speaking, if *P*^*X*^(*t*)is a predictable process, then it is “known” just ahead of time *t.* For a rigorous definition and some discussion see http://myyn.org/m/article/predictable-process/).

13 For a martingale *M*(*t*),*E*(*M*(*t* + *s*)|*X*[*t*, − *∞*]) = *M*(*t*) for all *t* and *s.* This states that the expected value of *M*(*t* + *s*) is that at time t, there is no “knowledge” (in the sense of expected value) for the future from the past, hence this type of process is taken as a representation of unpredictability.

14 This is a form of the famous Doob–Meyer decomposition of a stochastic process (Medvegyev, 2007).

17 Note that the interventional interpretation of DCM is motivated by the (temporal) asymmetry between the left- and the right-hand terms in Eq. (24). Its right-hand term gives us the expected rate of change $E\left[\dot{X}\left(t\right)\right]$ of *X*(*t*) if we fix *X*(*t*) to be *x* (i.e. if we perform the action *do*(*x*)), but does not provide any information about what *X*(*t*) is likely to be if we fix its rate of change $\dot{X}\left(t\right)$. This is best seen by noting that the system's motion $\dot{X}\left(t\right)$ is a proxy for the system's *future* state *X*(*t* + *Δt*), which cannot influence its own past *X*(*t*). Interestingly, this shows how interventional and prediction-over-time oriented (i.e. WAGS) interpretations of DCM are related.

18 The original motivation for the neural evolution equation of DCM for fMRI data considered the system's states *x* as being perturbations around the steady-state activity *x*_0_. Thus, *x* = 0 actually corresponds to steady (background) activity within the network (*x*_0_).

19 Interventional probabilities in a dynamical setting have recently been derived in, e.g., Eichler and Didelez (2010).

## Footnotes

4 With suggestion by A.N. Kolmogorov.

15 Roughly speaking *P*^*X*^(*t*) is measurable with respect to the process *X*_1_[*t*, − *∞*] and not *X*_2_[*t*, − *∞*] if all expected values of *P*^*X*^(*t*) can be obtained by integrating *X*_1_[*t*, − *∞*] without reference to *X*_2_[*t*, − *∞*]. The technical definition can be found in Medvegyev (2007). Basically this definition is based on the concept of a “measurable function” extended to the sets of random variables that comprise the stochastic processes.

16 In particular the Sinai–Ruelle–Bowen measure for hyperbolic dynamical systems (Chueshov, 2002).

20 Such a data set in an animal model including EEG, EcoG, DWI tractography and fMRI is being gathered by Jorge Riera (Tohoku University), within a collaboration including F. H. Lopes da Silva, Thomas Knoesche, Olivier David, and the authors of this paper. This data set will be made publicly available in the near future.
